# Efficacy of diagnostic testing for allergen sensitization in canine atopic dermatitis: a systematic review

**DOI:** 10.3389/fvets.2025.1551207

**Published:** 2025-05-21

**Authors:** Roger Morales-Romero, María S. Gonzalez-Dominguez, Jorge Sánchez, Nathalia M. Correa-Valencia, Juan G. Maldonado-Estrada

**Affiliations:** ^1^OHVRI Research Group, Facultad de Ciencias Agrarias, Universidad de Antioquia (UdeA), Medellín, Antioquia, Colombia; ^2^Grupo de Investigacion INCA-CES, Facultad de Medicina Veterinaria y Zootecnia, Universidad CES, Medellin, Colombia; ^3^Bivett Centro Veterinario, Medellin, Colombia; ^4^Group of Clinical and Experimental Allergy, Hospital Alma Mater de Antioquia, University of Antioquia, Medellín, Antioquia, Colombia; ^5^CENTAURO Research Group, Facultad de Ciencias Agrarias, Universidad de Antioquia (UdeA), Medellín, Antioquia, Colombia; ^6^Hospital Veterinario, Facultad de Ciencias Agrarias, Universidad de Antioquia (UdeA), Medellín, Colombia

**Keywords:** Allergens, canine atopic dermatitis, diagnostic methods, IDT, IgE, SAT, SPT, systematic review

## Abstract

**Introduction:**

Canine atopic dermatitis (CAD) is an important cause of quality of life in dogs and their owners. There are different diagnostic tools to evaluate sensitization to allergens in CAD; however, there is little information to evaluate the clinical usefulness of these tests.

**Methods:**

A systematic review aiming to assess the accuracy of allergen sensitization tests in CAD patients was performed. The search was planned, executed, and reported following PRISMA standards. The OVID®/MEDLINE, PubMed®, SciELO, and Redalyc databases were searched to find relevant studies comprising “diagnostic method” (OR test OR diagnosis OR method OR diagnostic OR paraclinic=) AND (atopic = OR hypersensitivity* OR allergen* OR “allergic reaction*” OR atopy) AND (dermatitis OR eczema OR scratching OR prurit = OR erythema OR rash OR edema) AND (canine OR dog* OR bitch* OR pupp*) search. Defined relevant articles were systematized, and content was analyzed via Atlas TI Scientific Software Development Software.

**Results:**

The groups of diagnostic tests included the intradermal test (IDT), serologic-specific allergen test (SAT), skin prick test (SPT), and patch test. Combining the results from all the search engines and deduplicate elimination, yielded 928 eligible citations published between 1963 and 2024, and the 72 articles that met the eligibility criteria were included in the qualitative synthesis evaluating SAT (*n* = 36), IDT (*n* = 37), SPT (*n* = 2), and patch tests (*n* = 1) reporting the use of 136 different allergens. Favrot’s clinical criteria were applied in 41.6% of the studies (30/72), with no previous consensus on the case definition for CAD.

**Discussion:**

The results of the review indicate that there is little information available to establish the diagnostic performance of the tests, which makes it difficult to make a recommendation regarding their use. In this systematic review they identified gaps in current knowledge that suggest the need for future research to standardize allergenic extracts, define cutoff points in serological tests, and consider environmental, geographic, and demographic variables. These findings provide a solid basis for improving the diagnosis and management of CAD and guiding future research in this field. Further studies are needed to adequately establish the diagnostic performance of the tests and their actual clinical usefulness.

## Introduction

The skin, the largest and most visible body organ, is a significant concern in veterinary consultation. Dermatopathies account for nearly 30% of general consultations in dogs (1). These conditions are often frustrating for both veterinarians and pet owners, as they impact an animal’s quality of life and appearance, which are particularly important to pet owners (2). Pruritus is the primary reason for dermatological consultations in dogs, with allergic conditions such as flea allergy dermatitis, food hypersensitivity, and atopic dermatitis being the most prevalent underlying causes (1). Pruritus (3) and several behavioral problems (4) account for the most critical findings affecting the quality of life of canine atopic dermatitis (CAD) patients.

Atopic dermatitis is a common condition in both humans (called AD) (5) and dogs (called CAD) (6, 7), with an estimated prevalence of 10–15% and a high tendency for relapse (8). This disease is defined as a complex, multifactorial inflammatory syndrome in which the skin is the primary exposure route. While similar and divergent pathophysiological mechanisms have been identified in humans and canines, dogs are frequently used as experimental models for studying human AD (9).

The primary underlying cause of AD appears to be a type-1 hypersensitivity reaction driven by mechanisms mediated by IgE. This mechanism is observed in humans (5) and 40–90% of canine cases (7, 10). Inflammation is triggered by normally innocuous environmental proteins and antigens, commonly called allergens (6, 8). An allergen triggers an exaggerated inflammatory response in susceptible individuals. This response leads to the production of IgE antibodies and the release of proinflammatory mediators, resulting in allergic symptoms such as itching, hives, and respiratory difficulties. Allergens are classified on the basis of similarities in their molecular structure and allergenic potential (11).

Reactivity to common epitopes grouped in allergen mixtures used in dermabrasion test extracts has been proposed. However, variations in the concentrations of individual allergens and their fractions in most allergen identification tests raise concerns about the reliability of these mixtures for diagnostic purposes and for the formulation of allergen-specific immunotherapies (12, 13). Co-sensitization can occur when an individual is sensitized to multiple allergens simultaneously, usually through cross-reactivity. This phenomenon arises when the immune system recognizes similar protein structures in related or unrelated allergens, provoking an inflammatory response.

The diagnosis of CAD is predominantly based on the widely recognized Favrot’s clinical criteria (14). These consist of two set of findings: Set 1: (i) Affected ear pinnae. (ii) Affected front feet. (iii) Age at onset <3 years. (iv) Chronic or recurrent infections (Mainly related to *Malassezia* yeast, and *Staphylococcus pseudintermedius*). (v) Corticosteroid-responsive pruritus. (vi) Mostly indoor. (vii) Non-affected dorso-lumbar area. And (viii) Non-affected ear margins. Set 2: (i) Affected front feet. (ii) Affected ear pinnae. (iii) Age at onset <3 years. (iv) Mostly indoor. (v) Non-affected dorso-lumbar area. (vi) Non-affected ear margins. And (vii) Pruritus sine material at onset (14).

This diagnostic approach requires meeting at least 5 out of 8 specified criteria (2), providing a sensitivity of 85% and a specificity of approximately 80% for identifying the syndrome. Notably, no significant predisposition has been reported regarding breed, age, or sex (15). Accordingly, the Favrot’s criteria are critical but not exclusive for CAD definitive diagnosis, for which Anamnesis, the CADESI score, and Pruritus Visual Assessment Score (PVAS), must be considered. Finally, diagnostic test must provide data to the most probable allergen the dog is allergic to.

Diagnostic tests for AD include methods such as dermabrasion to the epidermal level (prick test—SPT) or the dermal layer (intradermal skin test—IDT), which use a standardized panel of allergens to evaluate the patient’s reactivity against them (16). In dogs, IDT is the test of choice (17), whereas in humans, SPT is preferred because of its lower cost, faster interpretation, greater safety, higher specificity, and reduced pain (18). The serologic-specific allergen test (SAT), which measures specific IgE levels in the blood in response to common allergens, has also been documented and evaluated for CAD diagnosis. However, the results have been inconsistent, and there is no consensus on its reliability (17, 19). Another approach, the patch test, involves the epicutaneous application of allergens to assess cellular hypersensitivity to food and environmental allergens. This test aims to replicate the immune changes observed in natural lesions. However, its results remain controversial, particularly for food allergens (20, 21).

These diagnostic techniques have the potential to be useful for (1) supporting the clinical diagnosis of CDA, (2) establishing environmental restriction measures, (3) selecting extracts for allergen-specific immunotherapy, and (4) determining the prognosis of clinical control or remission. However, it is necessary to define the diagnostic performance of the tests for each outcome to establish their clinical utility (e.g., sensitivity, specificity, predictive values, and kappa index). Considering these challenges, our objective was to systematically compile and analyze the existing evidence from studies investigating the allergens utilized in CAD diagnosis, aiming to provide clarity and insights into this complex diagnostic process.

## Methods

This systematic review was planned, executed, and reported in accordance with PRISMA standards (22). The research question, methodology for conducting literature searches, study inclusion/exclusion criteria, and checklists for relevance screening, baseline characterization, methodological assessment, and data extraction from relevant primary research were all conducted on the basis of a preestablished and pretested protocol.

### Search strategy

Our goal was to evaluate the diagnostic performance of the available tests for CAD. The initial search took place on June 11, 2024. Four search databases (i.e., OVID®/MEDLINE, PubMed®, SciELO, and Redalyc) were searched. The topic was divided into components, and the search terms used to find relevant studies on the platforms were (“diagnostic method” OR test OR diagnosis OR method OR diagnostic OR paraclinic=) AND (atopic = OR hypersensitivity* OR allergen* OR “allergic reaction*” OR atopy) AND (dermatitis OR eczema OR scratching OR pruritus = OR erythema OR rash OR edema) AND (canine OR dog* OR bitch* OR pupp*).

### Eligibility screening

The inclusion criteria were limited to original articles published in peer-reviewed journals and written in English, Portuguese, French, or Spanish. The publication year and country of origin were not restrictive factors. The initial citation selection process involved evaluating the titles by two authors (RMR and MSGD), who selected citations on the basis of their potential relevance to the study topic. Two authors subsequently screened the list of citations chosen on the basis of their abstracts, following the inclusion and exclusion criteria established during the title screening phase. Afterward, two authors thoroughly reviewed the full texts of the remaining citations to ensure that they contained relevant data to address the research question. Kappa coefficients were calculated for each selection stage to assess agreement. Detailed scrutiny of each full text’s materials, methods, and results sections was conducted, with any conflicts resolved through author consensus. The World Association of Veterinary Dermatologists (WAVD) proceedings from 1989 to 2020 were available on its website.^1^ These proceedings were manually examined for any published primary studies. Additionally, as a final step, two authors manually searched the references cited in the pertinent articles identified during full-text screening, a process commonly referred to as snowballing, to uncover additional published sources.

To ensure eligibility, the articles defined as relevant were systematized, resulting in emerging categories and subcategories. A content analysis was developed via the Software Atlas TI Scientific Software Development GmbH (ATLAS.ti 24 Windows, 2022–2024). Subsequently, groups of diagnostic tests (i.e., IDT, SAT, SPT, and patch test) were assessed through an intentional coding analysis. Three recent articles were selected at the researchers’ discretion (23–25) to search for the most frequent concepts that would be considered trends. From there, the discriminated codes were obtained and served as a basis for the comprehensive review of the relevant articles. After all applicable publications were compiled, a descriptive summary was provided that considered the information of interest by groups of diagnostic tests.

### Risk of bias assessment

To assess the methodological quality of the included studies, a qualitative risk of bias evaluation was performed based on adapted domains from the QUADAS-2 tool, which is specifically designed for studies of diagnostic accuracy. The domains considered were: patient selection, index test, reference standard, and flow and timing. Two reviewers independently evaluated each study for potential sources of bias, and discrepancies were resolved by consensus.

## Results

The bias assessment revealed a high risk of bias in several domains across the included studies. Most studies (68/72) did not clearly define a reference standard for confirming CAD, increasing the likelihood of misclassification bias. Approximately 70% of the studies did not report blinding of test interpreters, leading to potential detection bias. Additionally, considerable variability in allergen concentrations and test protocols contributed to methodological heterogeneity. Only 41.6% (30/72) of studies applied Favrot’s clinical criteria, raising concerns regarding case definition and applicability. These findings suggest a moderate to high risk of bias in the body of evidence, which should be considered when interpreting the diagnostic value of allergen tests for CAD.

**Figure 1:**
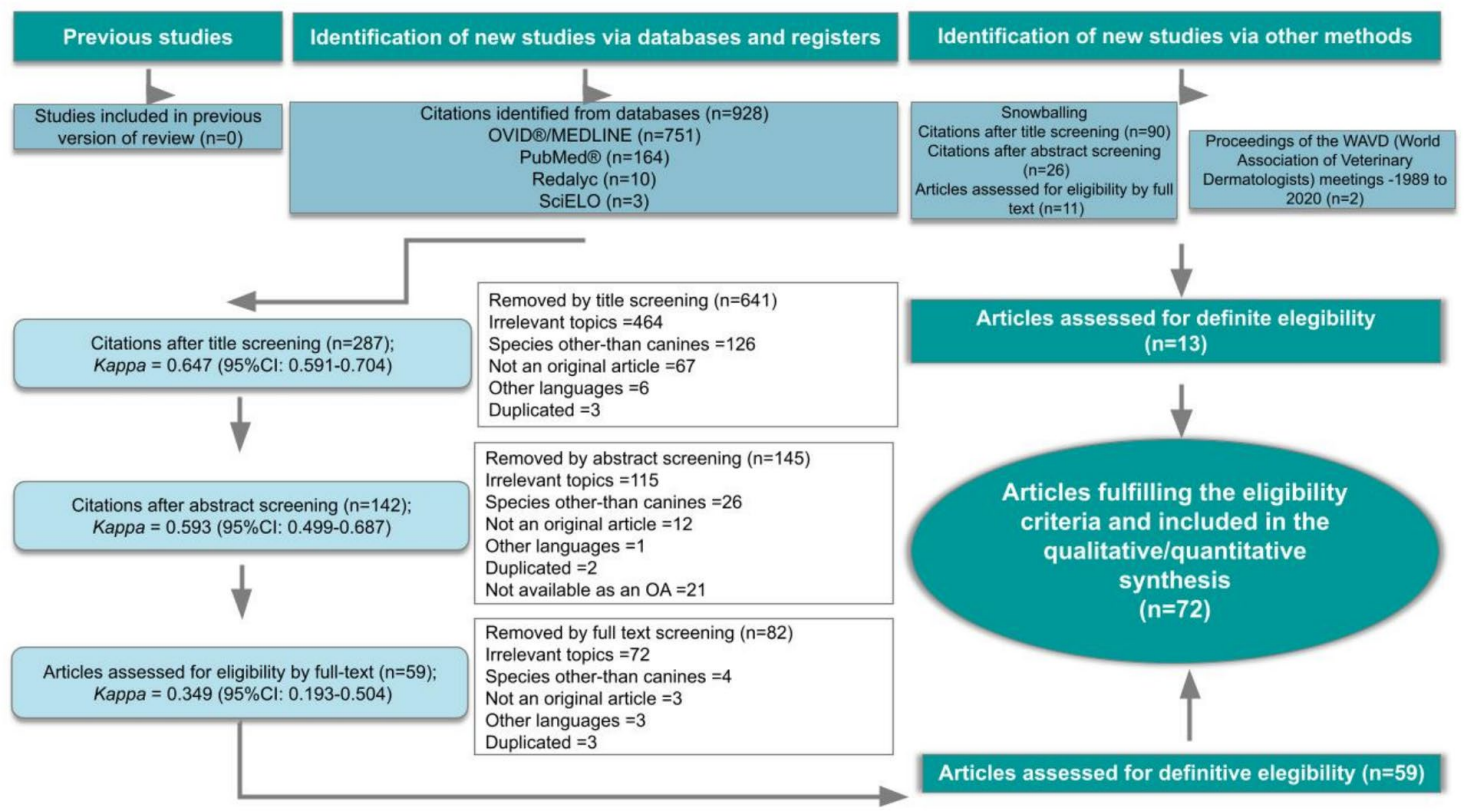
Preferred Reporting Items for Systematic Reviews and Meta-analyses (PRISMA) flow chart (22), which describes the progress of the citations through the systematic review. OA, Open Access.

The electronic search, which combines results from all search engines and, after deduplication, yielded 928 eligible citations possibly associated with the subject of this systematic review. The citations to be reviewed were published between 1963 and 2024. After the titles were read, 641 were considered unrelated (agreed upon by two reviewing authors). The final number of citations by title screening was 287 (retained by at least one reviewer). After the abstracts of the articles were read, 145 were excluded (by both authors), and 142 original articles remained for the full-text review. Sixty articles were reviewed in full text and kept for data extraction after 82 articles were dismissed during this phase. The snowballing strategy was applied through the reference lists of the 59 definitive articles, and 90 citations were retained after title screening. After the abstracts were screened, 26 studies were retained. The final selection of articles from the snowballing method yielded 11 results. In addition, two more articles were detected through the proceedings of the WAVD. The final number of articles that met the eligibility criteria and were included in the qualitative synthesis was 72. The file with the systematic process of collecting and selecting citations is available as Supplementary material (SM1). Figure 1 describes the protocol and the selection of relevant articles. All the articles were written in English. Studies were performed in the United States (*n* = 11), the United Kingdom, Japan, Brazil (*n* = 6, each), the Netherlands, Korea, Thailand (*n* = 4, each), Spain, Germany (*n* = 3, each), Austria, Norway, Poland (*n* = 2, each), Australia, China, Colombia, France and Italy (*n* = 1, each).

The first relevant article was published in 1982, and the most recent article was published in 2023. Citations were published in 31 journals and 18 countries, of which only three were nonseasonal countries (e.g., Brazil, Colombia, and Thailand). The results for the SAT, IDT, SPT, and patch tests corresponded to 36, 37, 2, and a single relevant study, respectively, reporting the use of 136 different allergens and considering relevant articles that addressed two or more diagnostic tests for CAD.

For SAT reports, 19 out of 36 articles (52.7%) reported positive reaction values in optical densities of variable cutoff points; 4 out of 36 (11.1%) reported in ELISA Units; 3 out of 36 (8.3%) used Top Screen and Immunodot values; and 10 out of 36 (24.7%) reported not reaction units (Table 1). For IDT reports, a high variability of cutoff point to define positive reactions was found, with a single report out of 37 (2.7%) reporting not positive reaction definitions (Table 2). Finally, for SPT reports, the two reports found reported different positive cutoff procedures and scales (Table 3).

**Table 1:** The study features when a serologic specific allergen test (SAT) was considered in the diagnosis of canine atopic dermatitis (*n* = 36).

Cons.	Ref.	Country	Sample size (n) and Sex	Breed (n)	Age	Favrot’s Criteria Yes/Not	Ig type: allergen type	Cutoff point to positivity	Relevant findings
1	(43)	The Netherlands	62 Males (30), females (32)	*Group A:* Boxer (10), German Shepherd (10), Spaniel (6), Terrier (6), Retriever (5). *Group B:* German Shepherd (7), Terrier (2). *Group C:* Boxer (11), German Shepherd (3), Scottish Shepherd (2)	*Group A*: 9 m-8.7 y (median: 2.5 y). *Group B*: 6–7.5 y (median: 2.9 y). *Group C*: 0.6–9 y (median: 3.5 y)	Not	House dust, human dandruff, dog dandruff, cat dandruff, and grasses	0 and 152 (OD) mean + 2 standard deviations (SD): 90.3 OD. IgGd +2 SD of the negative controls	There are ELISA and SAT discrepancies. There are no differences in specific allergen titters between allergens with similar polysaccharides (house dust and dander) and protein-rich allergens such as various pollen
2	(29)	The Netherlands	8 Males (3), females (5)	NR	1.5–8 y (median: 3.8 y)	Not	IgGd and IgE: DP (Der p I and Der p II) and DF (Der f I and Der f II)	±3 SD (≤0.150 OD positive)	No specific IgG was found for the Dep I, Der p II, Der f I, and Der f II fractions, which casts doubt on whether IgGd is a skin-sensitizing antibody in CAD patients
3	(44)	Spain	36 Sex NR	NR	NR	Not	IgE: Single: *Ctenocephalides felis*, DP y DF, *Alternaria* spp. *Aspergillus* spp., *Rhizopus* spp., kapok, Mucor, *Dactylis glomerata*, *Phleum pratense*, *Poa pratense, Festuca* spp., *Populus* spp., *Betula* spp., *Rumex acetosella, Plantago lanceolata, Artemisia* spp.*, Taraxacum* spp.*, Urtica* spp. Composed: house dust, cat dander, human dander, mixed feathers	OD >0.15 = positive	Sens: 72,23%. Spec: 41,6%. PPV: 76,47 and PNV 35,71%. False positive reactions were more frequent with elevated Sens and PNV. ELISA has limited value in distinguishing between CAD and nonatopic; it should not be used to confirm CAD. There is a poor correlation between IDT and ELISA
4	(38)	Japan	42 Males (22), females (20)	Mixed Breeds (7), Shiba Inu (4), Shetland Sheepdog (3), West Highland White Terrier (3), American Cocker Spaniel (2), Bichon Frise (2), French Bulldog (2), Golden Retriever (2), Great Pyrenees (2), Pug (2), and Akita, Bearded Collie, Dalmatian, Irish Setter, Japanese Terrier, Kai, Labrador Retriever, Maltese, Miniature Dachshund, Old English Sheepdog, Pomeranian, Siberian Husky, Shih Tzu (1 each).	3 m–9 y (median: 1 y)	Not	IgE: 24 allergens (not specified)	OD >0.3 = positive for the allergen group. OD >0.1 = positive for the specific allergen	HDM and Japanese cedar pollen were the most common allergens in CAD in Japan. Shiba Inu dogs with apparent predisposition to CAD
5	(49)	UK	265 Males (146), females (119)	Boxer, German Shepherd, Labrador, Golden Retriever, West Highland White Terrier (unspecified)	6 m–11 y	Not	IgE: Grasses: timothy grass, cocksfoot grass, meadow grass, rye grass. Weeds: sheep sorrel, plantain, nettle, lamb’s quarter, mugwort. Trees: alder, oak, birch. Molds: *Aspergillus* spp., *Penicillium, Cladosporium, Alternaria* spp. Mites*: Tyrophagus* spp., DF, DP, other allergens: Feather, cat epithelium, and flea	OD >0.250 = positive	Positive reactions in ELISA were equal to or greater than IDT for most allergens except HDM: DF (Sens 67.9%; Spec: 89.3%) and DP (Sens 19.3%; Spec: 96.6%). There is a strong correlation between IDT and DF. At 250 OD, the Sens for DF increased without loss of Spec, and DP increased with a slight reduction of Spec. The importance of using both tests together with clinical history and signs for CAD is clear
6	(26)	Norway	28 Males (11), females (17)	NR	1–8 y (median: 3.3 y)	Not	IgE: House dust, DP, DF, Flea, Human dander, Goose down, Cat epithelium, *Penicillium* spp., *Aspergillus* spp., *Alternaria* spp., *D. glomerata, F. elatior, L. perenne, P. pratense P. pratensis, B. verrucosa, Salix caprea, Populus* spp., *A. vulgaris, Taraxacum vulgare, P. lanceolata, Chenopodium album, R. acetosella, Urtica dioica*	OD >0.250 = positive	ELISA Sens 53.6% (low) and Spec 84.4%. PPV 75% and PNV 67.5%. There is no standardized cutoff for ELISA. The test does not measure allergen-specific functional IgE, which questions its usefulness. There was a poor correlation between IDT and ELISA in this study. The use of dust mite mixtures in ELISA led to low positive reactions
7	(51)	Spain	95 Males (70), females (28)	NR	9 m–11 y (median: 3.5 y)	Not	IgE: Single: DF, DP, and composed (a mix of *Lepidoglyphus destructor* and *T. putrescentiae*)	OD >150 = positive	91.6% of CAD dogs showed specific IgE to HDM in Galicia. They are considered important allergens in this and other humid and temperate regions
8	(52)	Germany	15 Sex NR	NR	1–11 y (median: 4.3 y)	Yes	IgE: DP, DF, *A. siro, T. putrescentiae, L. destructor, A. alternata, C. herbarum, A. fumigatus, Penicillium,* mixed grasses (Gramineae mixtum), bent grass (*Argostis alba*), Bermuda grass (*Cynodon dactylon*), Johnson grass (*Sorghum halepensis*), sorrel (*Rumex crispus*), English plantain (*P. lanceolata*), mugwort (*A. vulgaris*), lamb’s quarters (*C. album*), nettle (*U. dioica*), ragweed (*Ambrosia mixtum*), *Parietaria officinalis, Salsola kali*, birch (*Betula* spp.), alder (*Alnus* spp.), oak (*Quercus* spp.), cypress (*Cupressus* spp.), hazel (*Corylus avellana*), elm (*Ulmus campestris*), beech (*Fagus sylvatica*), poplar (*Populus* spp.) maple (*Acer pseudoplantanu*s), willow (*Salix* spp.), olive (*Olea europaea*) Flea saliva, cat dander (*Felis catus epithelium*), cockroach (*Blatella germanica*)	OD >150 = positive	Three laboratories with Fc-e receptor Allercept testing showed moderate intra- and interlaboratory variability, which was higher and clinically relevant with low OD values near the cutoff point. Although variability was acceptable in this study, a quality assurance program is needed to confirm reliability in veterinary medicine
9	(53)	Brazil	26 Sex NR	NR	NR	Yes	IgE: DF and *B. tropicalis*	OD was from 0.436–1.919	No serum from dogs with CAD recognized Der f 1 (a major allergen in humans). Groups 1 and 2 allergens were poorly recognized. Low molecular weight allergens such as Der f2 are considered minor in Brazil’s CAD. *B. tropicalis* allergens, commonly found in house dust in Brazilian cities, contribute to CAD
10	(54)	Norway	1,313 Males (673), females (640)	NR	0–6 m, >6 m–1 year (y), >1–2 y, >2–3 y, >3–4 y, >4–5 y, >5–6 y, >6–7 y, >7–8 y and >8 y.	Yes	IgE: *A. siro*, *T. putrescentiae*, and DF. timothy grass (*P. pratense*), cocksfoot (*D. glomerata*), annual bluegrass (*Poa annua*), perennial ryegrass (*L. perenne*), sheep sorrel (*Rumex* spp.), English plantain (*Plantago lanceolatum*), nettle (*U. dioica*), lamb’s quarter (*Chenopodium* sp.), mugwort (*A. vulgaris*), alder (*Alnus* spp.), oak (*Quercus* spp.), birch (*Betula* spp.), meadow fescue (*F. elatior*), velvet grass (*H. lanatus*), redtop grass (*Agrostis* spp.), elm (*Ulmus* spp.), beech (*F. sylvatica*), hazel (*Corylus* spp.), sycamore (*A. pseudoplatanus*), and ragweed (*Ambrosia* spp.), and flea saliva allergen	OD >150 = positive	There were no statistically significant differences between sexes for indoor allergens. Positive results were highest in dogs aged 1 to 4 years, and positivity decreased with age. No association was found between the season of birth and IgE results. Sampling during summer and fall showed higher positivity than in winter and spring. Boxers were the breed with the highest positivity. Norwegian CAD dogs were more likely to be hypersensitive to indoor allergens. Serum levels of allergen-specific IgE varied with sex, age, geographic location, and sampling season
11	(55)	Korea	101 Males (46), females (55)	Cocker Spaniel (15), mixed breed (13), Yorkshire Terrier (13), Shih Tzu (13), Maltese (12), Pekingese (4), Miniature Pinscher (4), Beagle (4), Dachshund (3), Miniature Schnauzer (3), and Pug, Cavalier King Charles Spaniel, Jindo Dog, Chihuahua, Bichon Frise (1 of each), unspecified (12)	5 m–13 y (median: 3 y)	Yes	IgE: 92 allergens not specified. Groups: grasses, trees, weeds, molds, epidermal allergens, house dust, foods, indoor allergens, insects, fleas, *Staphylococcus* spp., and *Malassezia* spp. allergens	OD >200 = positive	Sens 61.4% to HDM (similar to previous reports in same geographic area). HDM prevalence in this study was 55.2% (higher than previous research). This study found pollen (grasses, weeds, and trees) and molds commonly. Allergen-specific IgE serology may be valuable for CAD management
12	(56)	Poland	45 Males (24), females (21)	Labrador (7), German Shepherd (5), Short-haired Dachshund (5), Beagle (4), German Pointer (3), West Highland White Terriers (3), Boxer (2), Scottish Terrier (2), French Bulldog (2), Shih Tzu (2), and Poodle, Bulldog, Belgian Shepherd, Briard, Jack Russell Terrier, Fox Terrier, Airedale Terrier, Tibetan Mastiff, Central Asian Shepherd, Cocker Spaniel (1 of each)	1–8 y	Yes	IgE: Not specified	OD >150 = positive	In this study, Favrot diagnostic criteria (14) for CAD were used to evaluate the Sens and Spec of FcεRIα IgE ELISA for several allergens: D.F, Sens 82.3%. Spec 27.2%; D.P, Sens 65.1% and Spec 80%; A. Siro, Sens 89.4%. Spec 14.2%. IDT showed a lower error rate than ELISA (high sens on all allergens). The study confirms IDT as the gold-standard diagnostic method for CAD
13	(31)	Spain	18 Sex NR	Mixed breed (2) and other breeds (unspecified)	NR	Yes	IgE, IgG, IgG1, IgG2: DF: Der f1,2,18	OD > 0.35 = Positive	Sensitization to Der f 2 was higher than to Der f 1, with OD values ranging from 0.4 to 3.2. All the animals in our study were sensitized to both Der f 1 and Der f 2, although to a lesser extent, including healthy animals. Further studies with more serum samples and other allergens are necessary to confirm whether IgG plays a role in the pathogenesis of CAD. Additionally, all sensitized animals also recognized Der f 18
14	(58)	UK	59 Males (26), females (33)	Labrador and mixes (7), Pug and mixes (5), Cocker Spaniel and mixes (4), French Bulldog (3), English Bulldog (3), Hungarian Vizsla (3), Jack Russell Terrier (3), Cavalier King Charles Spaniel (2), Staffordshire Bull Terrier or its mixes (2), Rhodesian Ridgeback (2), unspecified (25)	Median: 2.95 y	Yes	IgE: Der f 2, Zen 1 and DF extract	OD cutoff: 0.161 for Der f 2, 0.247 for Zen 1, and 0.326 for *D. farinae*	The use of ELISA for recombinant Der f 2 demonstrated positivity for anti-Der f 2 IgE in 97% of cases and for Zen 1 specific IgE in 76%. Strong correlation between anti-Der f crude IgE and anti-Zen 1 IgE, with high IgE reactivity to the Zen 1 crude antigen
15	(60)	USA	596 Sex NR	NR	NR	Yes	IgE: Composed: allergen mixtures of mites, grasses, weeds, and trees	OD >150 = positive	Both carbohydrate-specific antibody inhibitors (RIDA-CCD) and bromelain-derived carbohydrate inhibitors (BROM-CCD) successfully reduced the reactivity of cross-reactive carbohydrate determinants (CCD), with BROM-CCD showing a more efficient inhibition profile. Reactivity to mite allergens in dogs and cats was not significantly affected, although a notable inhibition was observed for pollen allergens (trees, grasses, and weeds). After BROM-CCD inhibition, 1% of canine and 13% of feline samples evaluated negative for allergen reactivity. Using BROM-CCD may lead to more accurate allergen-specific IgE testing, thus excluding nonessential allergens from immunotherapy
16	(62)	Brazil	100 Males (44), females (56)	Lhasa Apso, Shih Tzu, Maltese, French Bulldog, mixed breed (unspecified)	11 m–14 y (median: 5 y)	Yes	IgE: DF, Der f 2, and native Zen 1 [following the methodology described by Chan et al. (63)]	OD > 0.2 = Positive	Seropositivity to Der f 2 (low molecular weight allergen) may be significantly higher. IgE OD values between D.F and Zen 1 were correlated but not with Der f 2. Zen 1 may cause sensitization to D.F. Frequent exposure to furniture and textile materials may promote sensitization
17	(40)	Korea	26 Sex NR	NR	NR	Yes	IgE: D.F and *T. putrescentiae*.	OD >0.25 = positive	The study found a higher prevalence of sensitization to Der f 18 in dogs with AD (84.6%) compared to a topic human (50%). Both humans and dogs showed high IgE reactivity to Der f 2 (86.7% in humans and 94.1% in dogs). However, both species exhibited low IgE binding to Tyr p 8
18	(24)	Brazil	85 Males (35), females (50)	Mixed breed (31), Shih Tzu (11), Lhasa Apso (8), Poodle (6), Yorkshire Terrier (4), Pinscher (4), German Spitz (2), and Pekingese, Maltese, Pitbull, Pug, Golden Retriever, English Bulldog, Staffordshire Bull Terrier, Dogo Argentino, Beagle, Boxer, Schnauzer, Pointer, Brazilian Terrier, Dachshund (1 of each).	1 y, <1 y, 1–3 y, 3–8 y, >8 y	Yes	IgE: DF, Der f 2, Zen 1	OD 0.196 for Der f2, 0.187 for Zen 1, and 0.299 for *D. farinae*	Canine IgE reactivity to *T. putrescentiae* was mainly due to nonspecific reactions and cross-reactivity with D.F. This finding highlights the different patterns of IgE sensitization between humans and dogs, particularly to Der f 18, suggesting the need for allergen standardization specific to each species
19	(65)	Portugal	68 Sex NR	NR	NR	Yes	IgE: Single: DF, DP, *A. siro, T. putrescentiae, L. destructor. D. glomerata, P. pratense. A. alternata, M. pachydermatis* Composed*: Aspergillus* mix	OD >150 = positive	House dust mites and storage mites were the most allergenic species. Significant correlations were observed between sensitization to different allergens. *M. pachydermatis* showed a high sensitization rate and was associated with severe dermatitis
20	(34)	UK	60 Sex not reported	Mixed breed (unspecified)	NR	Not	IgG and IgE: Single: DP, DF. Composed: insect mix	ELISA units (EAU) > 2 μg = positive	Immunoglobulin E (IgE) for *DP, DF*, and house dust was more frequent than IgG in CAD. IgG was more frequent for molds and insects than IgE in CAD, with allergen-specific IgG subclasses in CAD
21	(59)	USA	35 Males (18), females (17)	Mixed breed (11), American Pit Bull Terrier (5), Labrador Retriever (4), English Bulldog (3), Boxer (2), Golden Retriever (2), and Old English Bulldog, Chinese Shar Pei, Bull Terrier, Basset Hound, American Bulldog, Treeing Walker Coonhound, Cocker Spaniel, German Shorthaired Pointer (1 of each).	1–10 y (median: 4 y)	Yes	IgE: 49 allergens Not specified: fungi, grasses, insects, mites, trees, and weeds	EAU 0–79 = negative; 80–299 = positive; ≥300 = significantly positive	There are different methods for measuring allergen-specific IgE (such as pretest serum treatment, solid- or liquid-phase IgE capture techniques, canine IgE versus high-affinity IgE, and detection reagents using colorimetric versus radiometric methods), and there is no quality control of these tests. Confidence in allergen-specific IgE serology needs to be improved, as there were significant percentage differences when testing the same sample at the same time on different equipment and techniques
22	(61)	Italy	69 Males (44), females (25)	Labrador Retriever (11), French Bulldog (10), German Shepherd (8), American Staffordshire Terrier (6), mixed breed (6), English Bulldog (5), Rottweiler (4), Bernese Mountain Dog (3), Shih Tzu (2) Chihuahua (2), and Yorkshire Terrier, Shar Pei, Weimaraner, Pug, Border Collie, Chow Chow, Dalmatian, Australian Shepherd, Akita Inu, Staffordshire Bull Terrier, American Pit Bull Terrier, Scottish Terrier (1 of each)	2–10 y (median: 5 y)	Yes	IgE: Single: DF, DP, *A. siro*, *T. putrescentiae*, *L. destructor*, *A. alternata*, *Aspergillus fumigatus*, *Penicillium, Malassezia*, and flea antigens. Composed: *P. pratense*, *D. glomerata*, *P. pratensis*, *L. perenne*, *C. dactylon*, *T. vulgare*, *A. vulgaris*, *R. acetosa*, *P. lanceolata*, *Parietaria* spp., *C. album*, *Platanus acerifolia*, *O. europaea*, *Betula pendula*, and *Cupressus semperviren*.	EAU >150 = positive	High positivity was observed for storage mites and house dust mites. Elevated IgE in CAD from Northern Italy was associated with indoor allergens (HDM). Mold and flea saliva played a marginal role. Among outdoor allergens, *R. acetosa* (sorrel) played a significant role. Consider specific panels for geographical areas and assessments at regular intervals. Factors such as climate, pollination, plant distribution, environmental hygiene and residential environments affect IgE positivity in CAD
23	(33)	Thailand	44 Males (23), females (21)	Poodle (7), Shih Tzu (9), French Bulldog (5), Beagle (3), Pomeranian (3), mixed breed (6), Golden Retriever (3), Westy white terrier (2), and Shiba inu, Labrador retriever, Chihuahua, Jack Russel, American Pitbull, Bangkraw (1 of each)	Median: 6.62 y	Yes	IgE, IgG, IgG1, IgG2: Single: DF and DP	100 EAU, using the mean + 4 standard deviations (SD) from the mean of the negative control	Being older than 3 years was a risk factor for CAD. Healthy elderly dogs showed elevated levels of all isotypes (IgE, IgG, IgG1 and IgG2) against HDM. Both IgE and IgG1 were useful in distinguishing HDM-specific allergens in CAD. IgE and IgG1 showed better sens than IgG and IgG2 for HDM. IgG1 had similar clinical relevance as IgE in detecting HDM-specific allergens in CAD. A serological approach could serve as a valid and less invasive tool in CAD
24	(30)	Germany	66 Sex NR	NR	NR	Not	IgE: TOPSCREEN: A mix of grasses/rye, birch/oak/hazel, mugwort/plane tree, olive/*Parietaria* spp., Japanese cedar, HDM (DF and DP), storage mites (*Acarus siro*, *Tyrophagus putrescentiae* [TP]), epithelia: human dander, cat dander. Food A: cow’s milk, egg, soy, corn, wheat; Food B: beef, lamb, pork, fish mix, peanut. Molds: *Alternaria tenuis*, *Aspergillus fumigatus*, *Cladosporium herbarum*, *Penicillium notatum*, *Candida albicans.* ELISA: grass mix, rye, birch, hazel, mugwort, plane tree, *DF*, *DP*, *A. siro*, *T. putrescentiae*, *A. fumigatus*, *A. tenuis*	1 and 2 = negative, 3 = moderately positive, 4 and 5 = strongly positive	There was a good correlation between the TOP SCREEN group test and individual strips for indoor allergens, pollen, mold, and food allergens —very weak serological reactions, especially with insects
25	(45)	Austria	48 Sex NR	NR	NR	Not	IgE: House dust mites, storage dust mites, fleas, grass mix, tree mix (rye, birch, hazel, mugwort, plantain, alder), *Aspergillus* spp. *Alternaria alternata.* Cat epithelium. Food allergens (cow’s milk, egg, soy, bean, wheat, corn, lamb, beef, pork, fish mix, peanuts) 32 different allergens. Not specified	1–4 = positive, 0 = negative (no color reaction)	Sens and Spec varied by allergen; IDT showed higher values. There was a good correlation between IDT and Immunodot for HDM, not for other allergens. IDT is more reliable for diagnosing CAD, and Immunodot may be a complementary tool
26	(46)	Germany	84 Males (54), females (26)	Mixed breed (20), German Shepherd (11), Staffordshire Bull Terrier (6), Pitbull (2), Boxer (2), Labrador Retriever (2), Rottweiler (2)	1–10 y (median: 5,5)	Not	IgE: Cocksfoot (*D. glomerata*), Perennial ryegrass (*Lolium perenne*), meadow fescue (*Festuca elatior*), timothy grass (*P. pratense*), Kentucky bluegrass (*P. pratensis*), Yorkshire fog (*Holcus lanatus*). plantain (*P. lanceolata*), mugwort (*Artemisia vulgaris*), *Parietaria* spp. Flea: *C. felis*, birch (*Betula verrucosa*), oak (*Quercus alba*), hazel (*Corylus americana*), olive (*Olea europaea*). *A. tenuis, A. fumigatus, C. herbarum, Penicillium chrysogenum, C. albicans.* DF, DP	Positive control = 1+ − 4+; negative control = 0. Reactions were visually compared with controls using a color card (semiquantitative evaluation) and rated 0 = negative, and 1+, 2+, 3+, and 4+ = positive	CMG Immunodot (ELISA with allergen-specific anti-canine IgE monoclonal antibodies): Sens 90%. 86%. for HDM, fleas 68%. tree pollen 57%. grass pollen 75% and weed pollen 70%. for molds 0%. Overall Spec 81%. HDM 95%. fleas 91%. tree pollen 94%. grass pollen 92% and weed pollen 83%. molds 100%. High correlation (84%) between IDT and CMG Immunodot, high specificity and sens compared to IDT, being a useful test for CAD
27	(35)	USA	101 Sex NR	Foxhound, English Springer Spaniel, Soft-Coated Wheaten Terrier (unspecified)	NR	Not	IgG and IgE: NR	NR	IgE levels in dogs are higher than in humans and there does not appear to be a clear association between total IgE and CAD. IgG autoantibodies against IgE may affect serum IgE levels. IgE in helminth infections may lead to production of IgG against IgE epitopes
28	(28)	USA	NR Sex NR	NR	NR	Not	IgA: NR	NR	There are higher concentrations of IgA in the skin of CAD dogs than in normal dogs. This is not the case in serum
29	(36)	UK	65 Males (46), females (28)	NR	9 m–10 y (median: 4.3 y)	Not	IgGd and IgE: DF, DP, household dust, insects, molds, tree pollen, weeds, and grasses	NR	High positivity in HDM IDT in normal dogs, questions irritability at the concentration (1:10,000 w/v for DF and DP; 1,000 PNU for dust). ELISA and IDT positivity suggest heterogeneity of canine IgE. Spec, Sens, PPV, and PNV of IgE and IgGd are similar to dust mites. High levels of IgGd for DP in normal dogs compared to CAD suggest a protective function. The use of allergen mixtures is not recommended
30	(47)	Japan	16 Males (8), females (12)	NR	1–6 y (median: 3y)	Not	IgE: DF and DP, Der f 1, Der f 2, Der p 1, Der p 2	NR	Strong cross-reactivity between DF and DP in CAD. IDT and Immunodot ELISA are useful for the diagnosis of CAD. Sensitization to DF may be more predominant than to DP. Half of atopic dogs were sensitized to Der 1 and Der 2.
31	(48)	UK	33 Sex NR	NR	NR	Not	Ig E: DF, DP, Derp1, Der f1, Derp2 AP, Der f2	NR	Major allergens of *Dermatophagoides* were identified: a chitinase (Der f15), with no significant differences in Sens and Spec between polyclonal and monoclonal anti-canine IgE antibodies. Allergens of groups 1 and 2 of *Dermatophagoides* were not relevant in CAD. Good correlation between IDT and immunoblotting for DP and DF
32	(50)	USA	84 Sex NR	NR	NR	Not	IgE: *A. siro, B. tropicalis and T. putrescentiae,* HDM: DF, DP	NR	The concentration of IgE against HDM in our study is consistent with the high frequency of positive IDT. The variable geographic sens reflects diversity in the amount and type of exposure
33	(57)	Brazil	58 Sex NR	NR	6 m–8 y		IgE: DP, DF, *Blomia tropicalis, T. putrescentiae, A. siro, L. destructor.* Grass pollens: *C. dactylon, Phleum, Avena* (*Oat*), *Lolium,* Other pollens: *A. vulgaris, Chenopodium* spp., *Parietaria judaica, Ambrosia elatior, Betulaceae, Quercus*	NR	There was a high prevalence of sensitization to house dust mites (DF*, B. tropicalis*) and grass pollen (*C. dactylon*). The most common allergens in the IDT were *B. tropicalis* (48.5%), *C. dactylon* (42.4%), and D.F (33.3%). ELISA showed the highest sensitization to DF (86.5%), followed by *T. putrescentiae* (56.8%) and *B. tropicalis* (48.6%). No correlation was found between the results of IDT and ELISA
34	(37)	Japan	8 Males (4), females (4)	French Bulldog, Cavalier King Charles Spaniel, Mixed Breed, Maltese, Old English Sheepdog, Shih Tzu, Pekingese (unspecified)	1–11 y (median: 4.6 y)	Yes	IgE: 120 allergens (not specified)	NR	This study’s average concordance rate between SAT and IDT was 76.3%. Total IgE tests are unreliable and do not detect significant differences between normal and atopic dogs
35	(23)	Thailand	23 Males (11), females (12)	NR	4.3 ± 4.0 y	Yes	IgE: DF and *B. tropicalis*, *T. putrescentiae*, *A. siro*. Bermuda grass (*C. dactylon*), Bahia grass (*Paspalum notatum*), Johnson grass (*S. halepense*), meadow fescue (*Festuca pratensis*), timothy grass (*P. pratense*), red/curly dock (*R. acetosella*), lamb’s quarters (*C. album*), Russian thistle (*Salsola kali*), careless weed (*Amaranthus hybridus*), cocklebur (*Xanthium strumarium*), marsh elder (*Cyclachaena xanthiifolia*), common and giant ragweed (*Ambrosia artemisiifolia* and *A. trifida*), English plantain (*P. lanceolata*), mugwort (*A. vulgaris*), paperbark tree (*Melaleuca quinquenervia*), white mulberry (*Morus alba*), queen palm and oil palm (*Arecastrum romanzoffianum* and *Elaeis guineensis*), and white oak (*Q. alba*). *Malassezia pachydermatis* and flea saliva allergens	NR	Positive ASIS results for house dust were moderately to strongly associated with storage mites. House dust mites and weed pollen are the most significant sources of allergens in Thailand. During the rainy season, atopic dogs may experience higher IgE antibody titers. Cross-reactivity is likely between dust mite allergens and pollen allergens
36	(64)	Korea	189 Males (103), females (86)	Maltese, Bichon Frise, Poodle, Pomeranian, Mixed Breed, Shih Tzu, Yorkshire Terrier, Shiba Inu, Pug, French Bulldog, Dachshund, Spaniel, Golden Retriever, Welsh Corgi, Chihuahua, Husky, Spitz, Italian Grayhound (unspecified)	≤1 y, 2–3 y, ≥3 y	Yes	IgE: Composed: Baker’s yeast, egg white, egg yolk, milk, cheddar/gouda cheese, wheat, corn, rice, peas, soy, carrot, potato, sweet potato, pumpkin, tomato, apple, and peanut, pork, beef, duck, chicken, lamb, turkey, and venison, crab/shrimp, cod, tuna, salmon, and mackerel, cat epithelium, wool, and feather mix. Single: *P. notatum*, *C. herbarum*, *A. fumigatus*, *C. albicans*, *A. alternata*, and *M. pachydermatis*. Fleas, cockroaches, D.P, D.F, Acarus, *T. putrescentiae*, alder/birch, hazel, maple-leaf sycamore, willow/cottonwood, oak, white pine, acacia, and white ash, common ragweed, plantain, mugwort, sorrel, Bermuda grass, orchard/timothy grass, ryegrass, and cultivated rye	NR	A higher frequency was observed in males (54.8%) than females (45.2%). The highest prevalence (54.2%) was in dogs older than 3 years. The Maltese breed was the most affected (32.3%). Allergens were most frequently detected in fall (65.6%). Common allergens included: mold: *A. fumigatus* (95.6%) insects: fleas (31.9%) Food: corn (38.3%), potatoes (28.7%), duck (22.7%), Cod (24.4%) animal products: Wool (31.2%) trees: oak (41.8%), sorrel (25.0%)

AD, Atopic dermatitis; ASIS, Allergen-specific IgE serology; BU, Bethesda unit; CAD, Canine atopic dermatitis; DF, *Dermatophagoides farinae*; DP, *D. pteronyssinus*; EAU, ELISA absorbance units; IDT, Intradermal skin test; Ig, Immunoglobulin; Immunodot, Diagnostic test that uses allergen-specific strip tests to detect the presence of specific IgE antibodies in the serum; ELISA, Enzyme-linked immunosorbent assay; HDM, House dust mites; M, months; MWD, Mean wheal diameter; NE, Nitrogen equivalent; OD, Optical density; PBS, Phosphate buffered solution; PNU, Protein nitrogen unit; PNV, Negative predictive value; PPV, Positive predictive value; SPT, Prick test; SAT, Serologic specific allergen test; SD, Standard deviation; Sens, sensitivity; Spec, Specificity; w/v, weight to volume ratio; TP, *Tyrophagus putrescentiae*; y, Years; NR, Not reported; The samples were weighed per volume.

**Table 2:** The study features when an intradermal skin test (IDT) was considered in the diagnosis of canine atopic dermatitis (*n* = 37).

Cons	Ref.	Country	Sample (n) and Sex	Breed (n)	Age	Favrot’s Criteria Yes/Not	Allergen (concentration)	Cutoff point to positivity	Relevant findings
1	(66)	The Netherlands	15 (control) Sex NR	NR	NR	Not	Domestic dust allergen extract (0.01, 0.1, 1, and 10%), the domestic dust mite extract (0.1, 1, 10, and 100 U/mL), the human dander extract (0.01, 0.1, 1, and 5%), and the whole flea extract (0.025, 0.05, 0.1%. 0.2, and 0.4%)	Positive results: Papule diameter exceeded by at least 5 mm the diluent control	The maximum reaction occurred at 15 and 20 min. Threshold concentrations like those in humans: house dust extract (1%), house dust mite (10 U/mL), human dander (0.1%), animal dander (0.1%. 10-fold higher), pollen mixtures (1,000 Noon units/mL), fungi (1%), and fleas (0.1%). The commonly used pollen extracts of 1,000 to 1,500 PNU were too concentrated. The IDT is useful for diagnosing CAD. Wheal diameter is a reliable measure of assessment
2	(43)	The Netherlands	208 Males (104), females (104)	Boxer (28), Terrier (28), German Shepherd (27), Poodle (12), unspecified (113)	9 m–12 y (median:4y)	Not	The domestic dust allergen extract (0.01, 0.1, 1, and 10%), the domestic dust mite extract (0.1, 1, 10, and 100 U/mL), the human dander extract (0.01, 0.1, 1, and 5%), and the whole flea extract (0.025, 0.05, 0.1%. 0.2, and 0.4%)	Papule diameter exceeded by at least 5 mm the diluent control	Consideration of human dander in IDT for dogs. Lower incidence of reactions to mold compared to other studies. Multisensitivity to allergens was found in 59.1% with house dust, human dander and pollen
3	(66)	The Netherlands	62 Males (30), females (32)	*Group A:* Boxer (10), German Shepherd (10), Spaniel (6), Terrier (6), Retriever (5). *Group B:* German Shepherd (7), Terrier (2). *Group C:* Boxer (11), German Shepherd (3), Scottish Shepherd Dog (2)	*Group A:* 9 m–8.7 y (median: 2.5 y). *Group B:* 6–7.5 y (median: 2.9 y). *Group C:* 0.6–9 y (median: 3.5 y)	Not	The composition of the different mixtures has been reported previously (66). Each lyophilized allergen (10 mg)	Papule diameter exceeded by at least 5 mm the diluent control	There were discrepancies between immediate skin reactivity (IDT) and allergen-specific IgGa titers. This suggests that IgGa antibodies do not always coincide with immediate skin reactivity
4	(67)	UK	118 Males (63), females (55)	Labrador Retriever (56) West Highland Terrier (27), and Boxer (16) unspecified (19)	Median: 4.24	Not	DP, DF, *A. Siro*, and *T. putrescentiae* (10 NE/mL), house dust, human dander, horse epithelium, and mouse epithelium (10 μg/mL). cat epithelium: 10 NE/mL sheep epithelium, mixed feathers, American cockroach (500 PNU/mL), cotton litter: 350 PNU/mL, flea (*Ctenocephalides* spp.): 200 PNU/mL white ash (*Fraxinus Americana*), alder (*Ainus rugosa*), American hay (*Fagus americana*), white oak (*Q. alba*), white birch (*Betula populifolia*), American elm (*Ulmus americana*), black maple (*Acer negundo*) (1,000 PNU/mL). *Birch* spp., Western plantain (*Platanus occidentalis*), *Quercus* spp. (100 NE/mL). Kentucky grass (*P. pratensis*), fescue (*F. elatior*), orchard grass (*D. glomerata*), rye grass (*L. perenne*), sweet vernal grass (*Anthoxanthum odoratum*), timothy grass (*P. pratense*), velvet grass (*H. lanatus*), red grass (*Agrostis alba*) (1,000 PNU/mL). *Aspergillus mixture, A. tenuis, Botrytis cinerea, Epicoccum purpurascens, Penicillium* mixture, *Pullularia pullulans* (1,000 PNU/mL) *C. herbarum* (10 μg/mL). barley blight, oat blight, wheat blight: 500 PNU/mL	Positive result: the average papule diameter ≥ to the mean diameter of the histamine and diluent. A response of +2 or higher was considered positive Control diluent: graded as zero. Positive control (histamine) was graded as +4. Grading was from 0 to +4.	Allergen selection for diagnostic testing should consider environmental factors such as humidity and temperature on allergen sensitivity. The allergen panel evaluated in this study is valid for CAD in the UK. Further studies are needed to explore geographical variations in allergen prevalence
5	(29)	The Netherlands	8 Males (3), females (5)	NR	1.5–8 y (median: 3.8 y)	Not	Pure extracts at a concentration of 10 μg/mL. Purified fractions in 10-fold dilutions: Der p I at 18.25, 1.85, 0.18 μg/mL; Der p II at 1.37, 0.14, and 0.01 μg/mL; Der f I at 42, 4.2, and 0.42 μg/mL; Der f II at 1.2, 0.12, and 0.01 μg/mL	Positive: wheals between 50 and 75%: positive (1+); 75% of the diameter of Histamine solution were considered strongly positive (2+). Negative: < 1/2 of the diameter of the positive control	The fractions Der p I, Der p II, Der f I, and Der f II are less important for CAD. No specific IgGd was found for the fractions Der p I (25 kDa), Der p II (14 kDa), Der f I (25 kDa), or Der f II (14 kDa). This raises doubts about IgGd as a sensitizing antibody for the skin
6	(44)	Spain	36 Sex NR	NR	NR	Not	*C. felis*, DP, DF, mixed feathers, *Alternaria* spp.*, Aspergillus* spp., *Rhizopus* spp., kapok, domestic dust, cat dander, human dander, mucor, *D. glomerata*, *P. pratense*, *P. pratense*, *Festuca* spp., *Populus* spp., *Betula* spp., *Rumex acetosella, P. lanceolata, Artemisia* spp., *Taraxacum* spp., *Urtica* spp.	NR	Sens 72.23%. Spec 41.6%. PPV 76.47% and NPV 35.71%. ELISA has limited value in differentiating between atopic dogs and CAD. The correlation between IDT and ELISA was poor, with agreement in negative correlations (negative allergens in both tests). False positive reactions were more frequent than false negatives, high sensitivity, and negative predictive values
7	(36)	UK	65 Males (35), females (30)	NR	9 m–10 y (median: 4.3 y)	Not	Concentrations were based on the recommendations of the study by (66)	A + 1 to +4 scale based on erythema, turgor, papule elevation, and size (68) +1: barely perceptibly larger than negative control +4: closely approximated the size of the histamine papule	High positivity of IDT to HDM in normal dogs raises questions about the concentration (1:10,000 w/v for DF and DP; 1,000 PNU for house dust). The use of allergen mixtures without further purification is not recommended. There is a tendency for strong responses to IDT to show elevated levels of IgE. Positive IDT and IgE ELISA findings in normal dogs due to heterogeneity of canine IgE. Similar specificity, sensitivity, PPV and PNV of IgE and IgGd for HDM. Higher levels of IgGd to house dust in normal dogs suggest a protective role for this immunoglobulin
8	(30)	Germany	66 Sex NR	NR	NR	Not	Pollen and mold extract/1,000 PNU/mL; DP and DF at 1:1,000 and 1:5,000 w/v, respectively. Storage mite extracts: 100 PNU (NE)/mL	A + 1 to +4 scale based on erythema, turgor, papule elevation, and size (68) +1: barely perceptibly larger than negative control +4: closely approximated the size of the histamine papule	Sens (Positive = 1–2, 100%; Positive= >2, 54%), Spec (Positive = 1–2, 60%; Positive= >2, 93%), and Efficacy (Calculated concerning skin tests) (Positive = 1–2, 78%; Positive= >2, 76%). A good correlation was observed between the TOPSCREEN group assay and the individual strip for pollen, mold, and food allergens. Serological reactions were very weak, with weak IDT results. IDT tends to correlate with SAT
9	(45)	Austria	48 Sex NR	NR	NR	Not	House dust mites (10,000 square millimeter (SQ)/mL), grass mix, tree pollens (rye, birch, hazel, mugwort, plantain, alder; 100,000 SQ/mL), cat (10,000 SQ/mL), *Aspergillus* spp. and *A. alternata* (1:100 v/w) storage dust mites (*A. siro, Tyrophagus* spp., 500 BE/mL) and flea allergen (1,000 PNU/mL)	1+ reaction: erythema diameter 25% of the mean negative and positive controls. 2+ reaction: the mean value. 3+ reaction: diameters 25% larger than the mean value. The negative control was assessed as (−) 3+ reaction: positive control.	The IDT is considered more reliable for diagnosing CAD, while the Immunodot can be a complementary tool. Sensitivity and specificity varied depending on the allergen, with the IDT typically showing higher values. There was a good correlation between the IDT and Immunodot for dust mites, but not for other allergens
10	(46)	Germany	84 Males (58), females (26)	Mixed breed (20), German Shepherd (11), Staffordshire Bull Terrier (6), Pitbull (2), Boxer (2), Labrador Retriever (2), Rottweiler (2), unspecified (39)	1–10 y (median: 5,5)	Not	Plant allergens at 500 PNU/mL, dust mites were diluted to 1:75,000 w/v, and flea antigen to 1:1,000 w/v	Subjectively rated from 0 to 4, with strong positive reactions rated as 3 or 4.	Overall, ELISA Sens 90%. HDM 86%. fleas 68%. tree pollen 57%. grass pollen 75%. weed pollen 70%. molds 0%. Overall Spec 81%. HDM 95%. fleas 91%. tree pollen 94%. grass pollen 92%. weed pollen 83%. molds 100%. High correlation (84%) between IDT and CMG Immunodot. Overall Sens and Spec of CMG Immunodot were higher than IDT, being reliable to discriminate CAD and nonatopic patients
11	(47)	Japan	16 Males (8), females (12)	NR	1–6 y (median: 3 y)	Not	A DF and DP crude extract was prepared (69). Der f 1, Der f 2, Der p 1, and Der p 2 were purified from DF and DP cultures (70, 71) Concentrations of 1:20 or 1:50 were made with a diluent of 0.9% sodium chloride and 0.4% phenol (69)	Positive: size ≥ than the positive control (+++) Negative: ≤ than the negative control (−).	In atopic dogs, there is a strong cross-reactivity between DF and DP allergens. Sensitization to DF may be more predominant than DP, Sens to Der 1, and Der 2 at 50%. IDT and SAT (Immunodot and ELISA) methods are useful for the diagnosis of CAD
12	(72)	Greece	Males (45), females (46)	German Shepherd (18), French Poodle (10), Yorkshire Terrier (6), Cocker Spaniel (6), unspecified (51).	5 m–12 y (median: 3.8 y)	Not		Objective biases by the first two authors of the study (68, 73)	CAD is common in dogs in Greece, with a high prevalence of reactions to house dust mites, especially DF. No sexual preference was found, but certain breeds such as the Yorkshire Terrier, Chinese Shar-Pei, and Cocker Spaniel showed a higher predisposition to CAD
13	(38)	Japan	NR Sex NR	NR	NR	Not	Crude extract of Japanese Cedar pollen: 200 ng/mL	+++: reaction ≥ than the diameter of the positive control. ++: reaction ≥ than the average diameter of the positive and negative controls. +: Greater than the diameter of the negative control but smaller than the average diameter of the positive and negative controls. Negative: Equal to or smaller than the diameter of the negative control.	Dogs with CAD can develop a Type I hypersensitivity reaction to Japanese Cedar pollen allergen, similar to what is observed in humans. More studies are needed to determine its clinical relevance
14	(74)	Japan	95 Males (40), females (55)	Shih Tzu (20), crosses (12), Golden Retriever (11), West Highland White Terrier (11), Shiba Inu (9), Shetland Sheepdog (7), unspecified (20).	NR	Not	HDM mix, flea: 1:10,000 w/v, Cat epithelium, Grass mix, Mugwort, *Aspergillus* spp.*, Cladosporium* spp., *Penicillium* spp.: 1,000 PNU, Japanese cedar: 1:4,000 w/v	0 = wheal the same size as the negative control 1+ = one-quarter the size of the positive control 2+ = half the size of the positive control 3+ = three-quarters the size of the positive control 4+ = wheal the same size or larger than the positive control A positive reaction was rated as equal to or greater than a 2+ reaction.	IDT and SAT are useful for identifying allergens in CAD and immunotherapy formulations, with a high incidence of sensitivity to HDM (house dust mites) in Japan. There was good agreement between the results of IDT and SAT, although SAT showed lower sensitivity compared to IDT
15	(75)	USA	115 Males (78), females (49)	Labrador (26), mixed breed (24), Golden Retriever (9). unspecified (56)	9 m–10 y	Not	DP, DF: 1:50,000 w/v. Mixed HDM: 1:25,000 w/v (a 1:1 mixture of DP: DF Domestic dust extract: 25 PNU/mL)	Graded on 0 to 4 a scale (based on size, intensity of erythema, and turgidity). Positive: those reactions with a score of 2 (half the size and turgidity of the reaction induced by the histamine control solution) or higher.	The mixed extract of HDM showed false negative results. The house dust extract had low sensitivity and was not spec for HDM. Individual extracts of DF and DP are recommended instead of mixed or house dust extracts to avoid false negatives and reliability
16	(39)	Australia	1,000 Sex NR	NR	NR	Not	Most extracts at a concentration of 500 PNU/mL. HDM were analyzed at two dilutions: 1:7,500 and 1:75,000 w/v	Reactions were graded on a scale from 0 to 4 based on size, intensity of erythema (redness), and turgor (swelling). Positive reactions: scored as 2 or higher, meaning the reaction size and turgor were at least half of the size and turgor induced by the histamine control solution	HDM, particularly DF, are the most important allergens in CAD in southeastern Australia. Grass, weed, and tree pollen also play an important role. Plant species cross-react, and environmental factors affect pollen distribution
17	(38)	Japan	42 Males (22), females (20)	Mixed breeds (7), Shiba Inu (4), Shetland Sheepdog (3), West Highland White Terrier (3) American Cocker Spaniel (2), Bichon Frise (2), French Bulldog (2), Golden Retriever (2), Great Pyrenees (2), Pug (2), and Akita, Bearded Collie, Dalmatian, Irish Setter, Japanese Terrier, Kai, Labrador Retriever, Maltese, Miniature Dachshund, Old English Sheepdog, Pomeranian, Siberian Husky, Shih Tzu (1 of each)	3 m–9 y (median: 1 y).	Not	The concentration was generally 10,000 PNU/mL, DP and DF: 1:500 w/v. Japanese cedar pollen: 100 ng/mL.	+++: Diameter ≥ than the positive control. ++: Diameter ≥ the average diameter of the positive and negative controls. +: Diameter ≥ the negative control but smaller than the average diameter of the positive and negative controls	HDM (especially DF compared with DP) and Japanese cedar pollen are the most common allergens in CAD in Japan. Selection of allergens for IDT and SAT should be based on environmental factors and their geographic distribution. Shiba Inu dogs showed a breed predisposition to atopy
18	(48)	UK	33 Sex NR	NR	NR	Not	57 allergens, including DP, DF, Der p1, Der f1, Der p2, Der f2 (not specified)	0 to 4 compared to the controls. Positive: reactions rated ≥2.	This study demonstrated the recognition of major and minor allergens of *Dermatophagoides* by canine IgE. Major allergens identified included a chitinase, Der f15, with no differences in sensitivity or specificity between polyclonal and monoclonal anti-canine IgE. The *Dermatophagoides* group 1 and 2 allergens have limited relevance in CAD. There was a good correlation between IDT and immunotransfer for DP and DF
19	(49)	UK	265 Males (146), females (119)	Boxer, German Shepherd, Labrador, Golden Retriever, West Highland White Terrier (unspecified)	6 m–11 y	Not	DP, DF, flea: 1/10,000 w/v, *T. putrescentiae:* 100 NU/mL other allergens: 1,000 PNU/mL: timothy grass, cocksfoot grass, meadow grass, ryegrass. Sheep sorrel, plantain, nettle, lamb’s quarter, mugwort. Alder, oak, birch. *A., Penicillium, Cladosporium* spp., *Alternaria* spp., feather, cat epithelium, flea	Positive: wheal scoring 3 or 4 compared to the histamine positive control (score of 4)	The sensitivity of ALLERCEPT compared to IDT ranged from 6.3 to 77.1% for all allergens, and the spec ranged from 64.2 to 99.3%. There was a strong and highly significant correlation in IDT for DF and DP, but it was less strong in the ALLERCEPT panel. The ALLERCEPT panel showed higher positive reactions compared to IDT for most allergens. Careful validation of the cut-offs is essential to improve sensitivity without compromising specificity
20	(26)	Norway	28 Males (11), females (17)	NR	1–8 y (median: 3.3 y)	Not	DP, house dust, mixed feathers, *Penicillium* spp., *Aspergillus* spp.*, D. glomerata, F. elatior, L. perenne, P. pratense, P. pratensis, Betula* spp., *Salix* spp.*, T. officinale, P. lanceolata, C. album, Rumex acetosella, A. negundo*: 1,000 PNU/mL. DF and flea: 1:1,000 w/v	Positive: Wheal diameter ≥ the average between the diameter of the saline and histamine phosphate controls.	ELISA Sens 53.6% (low) and Spec 84.4%. PPV 75% and PNV 67.5%. There is no standardized cutoff for ELISA. The test does not measure allergen-specific functional IgE, which questions its usefulness. There was a poor correlation between IDT and ELISA in this study. The use of dust mite mixtures in ELISA led to low positive reactions (75)
21	(76)	USA	30 Males (14), females (16)	Mixed breed (12), Labrador Retriever (7), German Shepherd (3), Rottweiler (2), and Pitbull, Boxer, Doberman Pinscher, Border Collie, Jack Russell Terrier, Golden Retriever (1 of each)	2–8 y (median: 4.1 y)	Not	Flea allergen at 1:500, 1:750, 1:1,000, and 1:1,250 w/v. House dust mites and storage mites at 100, 250, 500, and 750 PNU/mL. Epithelia were analyzed at 500, 750, 1,000, and 1,250 PNU/mL, except for human dander, which was analyzed at 50, 100, 300, and 500 PNU/mL. The maximum available concentration for dander was 700 PNU/mL	Positive: if erythematous and, or indurated, and papule diameter ≥ than the mean diameter between the negative control and the positive histamine phosphate control (H1 or H2). Threshold concentration: the highest concentration at which 10% or fewer dogs (≤ 3/30 dogs) reacted positively.	Threshold concentrations for allergens: grasses, weeds, trees, molds, and insects (1,750 PNU/mL), Flea allergen (1:500 w/v), DP: 250 PNU/mL, DF and *T. putrescentiae* (100 PNU/mL) Epidermal, (all at least 1,250 PNU/mL), human dander (300 PNU/mL). histamine concentration (1:100,000 w/v) and 1,000 PNU/mL may be inadequate, resulting in false positives or negatives; the optimal concentration for IDT was 1:10,000 w/v
22	(82)	USA	26 Males (14), females (12)	NR	15 m–8 y (median: 4.1 y)	Not	The PNU/mL of *T. putrescentiae* extract tested were 1,000, 500, 250, 125, 63, 32, and 16.	*Objective evaluation:* Positive reaction: a diameter > half of the negative and positive control diameters’ sum. *Subjective evaluation:* A 0 to 4 scale was used; 0 = negative, and 4 = positive control or larger reaction. Positive reaction: subjective score ≥ 2.	The IDTs with *T. putrescentiae* extract do not differentiate between healthy dogs and dogs with AD. The most common positive reactions occurred at a concentration of 1,000 PNU/mL
23	(78)	Brazil	25 Sex NR	NR	12 m–11 y (median: 4.4 y)	Not	DP and DF: 0.4 μg/mL of Der p 1 and Der f 1, and 0.2 μg/mL of Der p 2 and Der f 2, equivalent to a biological activity of 1 BU/mL*. L. destructor:* 0.3 μg/mL of Lep d 1, corresponding to 1 BU/mL. *B. tropicalis* and *T. putrescentiae*: biological activity of 1 BU/mL.	Positive: Wheal diameter ≥ than the average diameter of the histamine and negative reaction.	Although the extracts are standardized for humans, they may be effective in CAD. The most frequent positive reactions in CAD were to T*. putrescentiae* and *L. destructor* (importance of including them in IDT). There was a significant difference in the prevalence of positive reactions in dogs with CAD and healthy dogs
24	(83)	Thailand	114 Males (63), females (51)	Poodle (24), mixed breed (24), Golden Retriever (17), Shih Tzu (16), Labrador Retriever (6), Thai Bangkaew (6), Pug (5), Beagle (3), Chow Chow (3), Bull Terrier (2), German Shepherd (2), West Highland White Terrier (2), and French Bulldog, Akita, Rottweiler, Thai Ridgeback (1 of each).	9 m–11y (median: 3.6 y)	Not	HDM mixed (100 PNU/mL), DF, DP (100 AU/mL), domestic dust and mixed ants, mixed cockroaches, housefly, kapok (1,000 PNU/mL) pollen (grass = G, weed = W, tree = T): fire ant, mosquito: 1:1,000 (w/v), American cockroach, German cockroach: 1:200 (w/v) para grass (G), Bermuda grass (G), timothy grass (G) (1,000 BU/mL), Bahia grass (G), Johnson grass (G), Wild oats (G), amaranth (W), spiny pigweed (W), common wormwood (W), burdock (W), goldenrod (W), cowslip (W), sorrel (W), kochia (W), eucalyptus (T), royal palm (T), Australian pine (T), acacia (T), red mulberry (T), white mulberry (T): (1:200 w/v), Sweet corn (G), wheat grain (G) (1:100 w/v), rush (W), typha (W) (1,000 PNU/mL). Mixed mold, *Cladosporium* spp., *A. fumigatus,* mixed *Aspergillus*, mixed *Penicillium*, *Alternaria* spp., *Acremonium* spp., *Curvularia* spp., *Drechslera* spp., *Fusarium* spp., yeast (1:100 w/v)	Positive reaction: the diameter and redness of the wheal ≥ to the mean diameter and redness of the papule induced by histamine and saline solution.	Prevalences: DP 74.56% and DP 53.51%: These are the most frequent allergens in dogs and humans in Thailand, with DF being more common in dogs and DP in humans. Other common allergens were house dust (26.32%), American cockroach (23.68%), Para grass (21.93%), and mixed ants (20.18%). No association was found between allergens and the location of skin lesions.
25	(84)	China	71 Males (47), females (24)	Golden Retriever (15), mixed breed (14), Beagle (8), Labrador Retriever (6), Maltese Terrier (6), Short-haired Dachshund (4), Miniature Poodle (3), Shiba Inu (3), Chihuahua (2), and Basset Hound, Bull Terrier, Chow Chow, Cocker Spaniel, French Bulldog, German Shepherd, Pyrenean Mountain Dog, Shetland Sheepdog, Shih Tzu, Welsh Corgi (1 of each)	8 m–12 y (mean: 2.9 ± 2.1 y)	Not	Cat epithelium, German cockroach preparation, house dust, human dander (not specified), Mixed dust mites (DP. DF), mixed domestic insects (flies, moths, American cockroaches, and mosquitoes) (100–250 PNU/mL or 1:1,000 w/v). *M. pachydermatis* extracts (1,000 PNU/mL). *Staphylococcus aureus* (120–180 UFC/mL)	Positive reaction: if rated with a score of ≥2 on a 0 to 4 scale.	House dust is the most common allergen in CAD (72%). DF (70%), D.P (70%), HDM mixture (68%), and insect mixture (33%). Correlation between reactions to house dust and HDM mixture, between DF and DP. Males were more susceptible to CAD than females. The most affected breeds were golden retrievers, crossbreeds, beagles, Labrador retrievers, and Maltese terriers. It is important to identify specific allergens and understand the environmental factors of CAD
26	(79)	USA	34 Males (16), females (18)	Mixed breed (12), Labrador Retriever (5), Cocker Spaniel (3), Pug (2), Basset Hound, Boxer, English Bulldog, Shih Tzu, Beagle, Yorkshire Terrier, Bichon Frise, American Staffordshire Terrier, Miniature Schnauzer, German Shepherd, Golden Retriever, Miniature Poodle (1 of each).	1–13 y (median: 4.8 y)	Yes	histamine (1:100,000 w/v) and a 0.9% saline solution. Most allergens were assessed at 1,000 PNU/mL. House dust (HDM mixture) at (1:100 w/v, 1:5,000 w/v and 1:10,000 w/v). House fly, flea, black ant and mosquito (1,000 PNU and 500 PNU), flea (1:4,000 w/v). Sheep epithelium (500 PNU and corn at 250 PNU)	Positive reaction: if rated with a score of ≥21 on a 0 to 41 scale (based on the perceived diameter of the wheal, erythema, and induration), or if the objective measurement of the wheal diameter was ≥ to the average of the positive and negative controls	The objective score can serve as a reference for individuals without experience. Combining subjective and objective methods can provide more reliable IDT results
27	(85)	Poland	142 Males (70), females (72)	German Shepherd (26), mixed breed (20), American Staffordshire Terrier (16), Boxer (14), Dogue de Bordeaux (10), Labrador (5), French Bulldog (5), Dachshund (4), Doberman (4), English Bulldog (3), Fox Terrier (3), unspecified (32)	1–6 y (median: 2.8 y)	Yes	Grass pollen mix: tree pollen mix I, tree pollen mix II, weed pollen mix, flea (1,000 NU/mL) *T. putrescentiae,* DF, DP, *L. destructor*, A. *siro*: (100 NU/mL), cat epithelium, fungi mix I (100 μg/mL) positive control (0.1 mg/mL histamine) and negative control (0.9% saline solution)	*Subjective assessment*: Positive = a wheal with erythema comparable to the positive control (histamine solution). *Objective assessment*: Positive reaction: a diameter ≥ than the mean value of the positive and negative controls. ++++: Diameter ≥ than the positive control (>14 mm). +++: Diameter smaller than the positive control (12–14 mm). ++: Diameter half the positive control (8–11 mm). +: Diameter > than the negative control by 3 mm but less than half the positive control (5–7 mm). –: Diameter ≤ than the negative control	The most frequent reactions to HDM (70.61%), particularly, are DF (93.66%). Pollen allergens (19.55%), especially in the grass pollen mixture. Animal allergens (4.15%) affect the cat epithelium. Mold allergens (1.66%) less frequent. Flea allergens (4.03%). Polysensitization in 98.6%
28	(77)	Korea	58 Males (30), females (26)	Maltese (11), Shih Tzu (11), Yorkshire (8) Terrier (8), Cocker (6) Spaniel (5), Pekingese (5), mixed breed (5), Pug (3), and Pomeranian, King Charles Spaniel, Schnauzer, French Bulldog, Poodle, Miniature Pinscher, Chihuahua, Fox Terrier, Sabsari (1 each)	1–13 y (mean: 4.8 y)	Yes	*Rhizopus* spp. (mold) mix (250 PNU/mL), house dust: (100 PNU/mL), HDM (DP, DF) (1:5,000 w/v) Fleas: 1:1,000 w/v, pollen, weeds, trees and shrubs, flowers, mold, coal, house dust, cat dander, cottonseed, kapok seed, pyrethrum, mixed silk and feathers, mosquitoes, two cockroach and flea mixes (1,000 PNU/mL)	Positive reaction: a diameter ≥ than half the diameter of the positive and negative controls’ diameter. *Subjective*: A scale from 0 to 4 was used to evaluate the wheal’s diameter, height, firmness, and erythema. 0: Negative control 4: Positive control Scores of 3 and 4 = positive and clinically relevant.	Mold, house dust, HDM, insects, and inhalants are common allergens for CAD in Seoul. Indoor lifestyles and climate changes in Korea may influence these findings
29	(41)	UK	651 Sex NR	NR	NR	Yes	DF, DP, A. siro, *T. putrescentiae, L. destructor* (0.001 w/v), human dander, fleas, cotton lint (0.0001 w/v), cat epithelium, sheep epithelium, mixed feathers, mixed cockroaches, mixed moths, mosquito (500 PNU/mL), white ash, alder, American beech, red oak, white birch, privet, western sycamore, willow, red cottonwood, poplar, cottonwood, weed pollens, sorrel, ragweed, goldenrod, viper’s grass, dandelion, angelica, mugwort, nettle, greater plantain, *Brassica* spp., *Chrysanthemum* spp., red clover, *P. pratensis*, *F. pratensis, D. glomerata, Lolium eterna*, *A. odoratum*, *P. pratense*, *H. lanatus*, *Agrostis* spp., *C. dactylon*, Oats, Wheat, *Aspergillus* spp., *A. tenuis, B. cinerea, Penicillium* spp., 1,000 PNU/mL, grain charcoal (1,000 PNU/mL)	Grading scale: 0 to 4 based on the size of the wheal, turgor, and erythema compared to the control sites. Positive reaction: test sites scoring ≥2 Negative: test sites scoring 0 or 1.	Most allergens within related groups (house dust/storage mites, epidermis/fibers, trees, weeds, grass pollen, and molds) showed statistically significant associations, indicating potential cross-reactivity or cosensitization. These findings suggest that related allergens may share common epitopes, which could influence allergen selection for testing and immunotherapy. Further research is needed to clarify these findings
30	(37)	Japan	8 Males (4), females (4)	French Bulldog, Cavalier King Charles Spaniel, mixed breed, Maltese, Old English Sheepdog, Shih Tzu, Pekingese (unspecified)	1–11 y (median: 4.6 y)	Yes	53 allergens (100 ~ 1,000 PNU/mL or 1:1,000 w/v). Some of the allergens are listed below: DT, DP, Mite mixture 1 Korea (DP, DF, D. microceras), House dust, *A. alternata, B. cinerea*, Mold mixture (*A. fumigatus*, *A. niger*, *A. amstelodami*, *A. nidulans*), Tree pollen mixture (cedar, cypress, juniper) Positive control: Histamine phosphate (27.5 μg/mL) Negative control: 0.9% saline solution with 0.1% phenol	+1: Wheal size > than negative control, < than the mean value between positive and negative control. (+2): Size > than the mean value between positive and negative control, and < than positive control. (+3): size is > than positive control. (−): Wheal size ≤ than negative control.	This study’s average concordance rate between SAT and IDT was 76.3%. Total IgE tests are unreliable and do not detect significant differences between normal and atopic dogs
31	(56)	Poland	45 Males (24), females (21)	German Shepherd (5), German (3) Pointer (7) Labradors (5), short-haired (5) Dachshund (2) Boxer (4), Beagle (4), West Highland White Terrier (3), Scottish Terrier (2), French Bulldog (2), Shih Tzu (2) and Poodle, Bulldog, Belgian Shepherd, Briard, Jack Russell Terrier, Fox Terrier, Airedale Terrier, Tibetan Mastiff, Central Asian Shepherd, Cocker Spaniel (1 of each)	1–8 y	Yes	DF, DP, *T. putrescentiae*: *A. siro*: 1:1,000 w/v Fosfato de histamina: 0.0275 mg/mL	Authors evaluated size and quality of the skin reaction. Reactions were graded on a scale of 0 to 4, where 0 represents a negative result and 1 to 4 represents positive results of varying intensities	Diagnostic criteria for CAD, according to Favrot, were used to evaluate the Sens and Spec of the FcεRIα IgE ELISA test. Results: DF, Sens 82.3%. Spec 27.2%. DP, Sens 65.1%. Spec 80%. *T. putrescentiae*, Sens 74.3%. Spec 33.3%. *A. siro*, Sens 89.4%. Spec 14.2%. IDT has a lower error rate than ELISA (high Sens in all allergens). IDT is the gold standard diagnostic method for CAD
32	(57)	Brazil	58 Sex NR	NR	6 m–8 y	Yes	DP, DF, *B. tropicalis, C. dactylon, P. notatum* (not specified).	Skin reactions were measured by calculating the average between the largest diameter and its perpendicular. If this average exceeded the positive control, it was considered a positive reaction	High sensitization to HDM (DF, *B. tropicalis*) and grass pollen (*C. dactylon*). The most common allergens in the IDT were *B. tropicalis* (48.5%), *C. dactylon* (42.4%) and DF (33.3%). ELISA showed the highest sensitization to DF (86.5%), followed by *T. putrescentiae* (56.8%) and *B. tropicalis* (48.6%). No correlation was found between the results of the IDT and the ELISA
33	(42)	France	261 Males (121), females (140)	Labrador Retriever (35), Boxer (19), West Highland White Terrier (16), French Bulldog (16), unspecified (175)	1–12.5 y (median: 5 y)	Yes	Timothy grass (*P. pratense*), Perennial ryegrass (*L. perenne*), Common meadow grass (*P. pratensis*), Mixed grasses: cocks’ foot (*D. glomerata*), Timothy grass (*P. pratense*), sweet vernal grass (*A. odoratum*), velvet grass (*H. lanatus*) and Bermuda grass (*C. dactylon*) (1,000 w/v)	Objective methods: measuring the diameter of the wheal. Positive = wheal diameter > than the average of the negative and positive controls (histamine) or at least 3 mm > than the negative control Subjective methods: presence of erythema and digital palpation. 0: negative reaction 3+: positive reaction.	No correlation was found between grass pollen sensitization and the dogs’ sex, age, or month of birth. Dogs sensitized to grass pollen often reacted to many other allergens, particularly DF. The accumulation of aeroallergens indoors could explain the lack of seasonality in sensitization
34	(23)	Thailand	82 Males (51), females (31)	Shih Tzu (11), Chihuahua (7), Pug (5), mixed breed (4), Poodle (3), Papillon (2), Dachshund (2), and Jack Russell, Schnauzer, and Yorkshire Terrier (1 of each). 17 Beagles, four mixed breeds, 5 French Bulldogs, 2 Siberian Huskies, and one each of the following: Shar Pei, Thai Ridgeback, Thai Bangkaew, Weimaraner, Welsh Corgi, German Shepherd, 6 Labradors, and 5 Golden Retrievers.	11 m–11 y (mean: 5 y)	Yes	DF, DP: 100 PNU/mL, mixed mites: 100 PNU/mL (prepared with 50 PNU/mL of DF and 50 PNU/mL of DP), house dust: 1,000 PNU/mL	A scale from 0 to 4+ was used, based on the size of the erythematous wheal 15 min after injection. Reactions were compared to the mean diameters and erythema of the positive and negative controls. A reaction was considered positive if it scored >2+	Prevalence: DF 64.63%. DP 58.54%. Mixed mites 47.56%. House dust 35.37%. There is a moderate correlation between DP and house dust (r = 0.514), DP and DF (r = 0.426), and DF and mixed mites (r = 0.423). Mixed mites: Sens 60.32%. Spec 94.70%. House dust: Sens 42.90%. Spec 89.50%. No significant differences were found in the *in vitro* results based on sex, breed, age, or coat length. HDM is an important source of allergens in CAD. Mixed mite extracts did not elicit skin responses as strong as individual extracts
35	(86)	Thailand	16 Males (10), females (6)	Beagle, Poodle, mixed breed, West Highland White Terrier, French Bulldog, Shih Tzu, Jack Russell Terrier, Labrador Retriever, Thai Ridgeback, Pug (unspecified)	1–15 y (median: 6.56 y)	Yes	There were 47 local aeroallergens, including Der f and Der p extracts (not specified)	Positive reaction: the diameter and redness of the wheal at the test site were ≥ to the average diameter and redness of the wheals at the positive control (histamine) and negative control (saline solution) sites	Five allergens were identified as major IgE-binding components to DF (Der f Alt a 10, EF1-*α*, the gelsolin-like allergen Der f 16, Der f 28, and Der f 2) and Der f 3, Der f 10, Der f 20, and Der f 32 as minor allergens. Cross-reactive invertebrate components such as α-enolase, serine protease, and arginine kinase were suspected
36	(33)	Thailand	44 Males (23), females (21)	Shih Tzu (9), Poodle (7), mixed breed (6), French Bulldog (5), Beagle (3), Pomeranian (3), Golden Retriever (3), Westy white terrier (2), and Shiba Inu, Labrador retriever, Chihuahua, Jack Russel, American Pitbull, Bangkraw (1 of each)	Median: 6.62 y	Yes	DF and DP, 40 μg/mL and 36.75 μg/mL, respectively	Positive reaction: if the diameter and redness of the wheal at the test site were ≥ to the average diameter and redness of the wheals at the positive control (histamine) and negative control (saline solution) sites	Being older than 3 years was a risk factor for CAD. Healthy elderly dogs showed elevated levels of all isotypes (IgE, IgG, IgG1 and IgG2) against HDM. Both IgE and IgG1 were useful in distinguishing HDM-specific allergens in CAD. IgE and IgG1 showed better sensitivity than IgG and IgG2 for HDM. IgG1 had similar clinical relevance as IgE in detecting HDM-specific allergens in CAD. A serological approach could serve as a valid and less invasive tool in CAD
37	(65)	Portugal	68 Sex NR	NR	NR	Yes	Single: DF, DP, *A. siro, T. putrescentiae, L. destructor. D. glomerata, P. pratense. A. alternata, M. pachydermatis* Composed: *Aspergillus* mix	Positive reactions: when the wheals were ≥ than half the size between the negative control (saline solution) and the positive control (0.01% histamine phosphate solution). Reactions were scored on a scale from 0 (negative) to 4 (maximum positive)	House dust mites and storage mites were the most allergenic species. Significant correlations were observed between sensitization to different allergens. *M. pachydermatis* showed a high sensitization rate and was associated with severe dermatitis

AD, Atopic dermatitis; ASIS, Allergen-specific IgE serology; BU, Bethesda unit; CAD, Canine atopic dermatitis; DF, *Dermatophagoides farinae*; DP, *D. pteronyssinus*; EAU, ELISA absorbance units; IDT, Intradermal skin test; Ig, Immunoglobulin; Immunodot, Diagnostic test that uses allergen-specific strip tests to detect the presence of specific IgE antibodies in the serum; ELISA, Enzyme-linked immunosorbent assay; HDM, House dust mites; M, months; MWD, Mean wheal diameter; NE, Nitrogen equivalent; OD, Optical density; PBS, Phosphate buffered solution; PNU, Protein nitrogen unit; PNV, Negative predictive value; PPV, Positive predictive value; SPT, Prick test; SAT, Serologic specific allergen test; SD, Standard deviation; Sens sensitivity; Spec, Specificity; w/v, TP, *Tyrophagus putrescentiae*; y, Years; NR, Not reported; The samples were weighed per volume.

**Table 3:** The studies featured when the prick test (SPT) was considered in the diagnosis of canine atopic dermatitis (*n* = 2).

Cons.	Ref.	Country	Sample size (n) Sex	Breed (n)	Age	Favrot’s criteria	Allergen (concentration)	Cutoff point to positivity	Relevant findings
1	(80)	Colombia	9 Sex NR	English Bulldog, French Bulldog, Beagle, West Highland White Terrier, Springer Spaniel, Maltese, Cocker Spaniel, Labrador Retriever, Yorkshire Terrier (1 of each). Control group: mixed breed (4), Standard Schnauzer (2), and Afghan Hound, Pitbull, Bull Terrier (1 of each)	4.2 ± 1.0 y	Yes	*Alternaria* spp. (3 μg/mL), *A. fumigatus* (25 μg/mL), *A. vulgaris* (50 mg/mL), *C. arizonica* (10 mg/mL), grass mix (*P. pratensis, D. glomerata, L. perenne, P. pratense, F. pratensis, Helictotrichon pretense*), *C. dactylon* (50 mg/mL) DF, DP (100 mg/mL), *B. tropicalis* (150 mg/mL), fire ant (1:100 w/v), cat epithelium (10,000 BU/mL), histamine (Positive Control: 10 mg/mL), diluted glycerol-saline solution (negative control: 1:20 w/v)	Positive reaction (objective): wheal diameter ≥ than 3 mm, using a metric ruler. It represented the minimum average between the diameters of the wheals from the positive and negative controls.	Sens: 66% (95%CI: 29.9–92.5%) Spec: 100% (95%CI: 66.3–100%) The SPT demonstrated high Spec and could be a valuable tool for diagnosing CAD. However, further research is needed in a larger population. House dust mites, such as *B. tropicalis*, DF, and DP, were the most common allergens eliciting reactions. PST does not require sedation, is less expensive, and is more specific than IDT
2	(25)	USA	40 Males (26), Females (14)	Mixed breed (14), Labrador Retriever (4), German Shepherd (3), Newfoundland (3), American Pit Bull Terrier (2), English Bulldog (2), Golden Retriever (2), and Australian Shepherd, Beagle, Boxer, Cane Corso, Chesapeake Bay Retriever, Great Dane, Husky, Pug, Shar Pei, and Shiba Inu (1 of each)	1–10 y (median: 4 y)	Not	DF, DP (SPT 1:400 w/v; IDT: 1,000 PNU/mL), mold mix 1: *A. alternata, Aspergillus niger, Bipolaris sorokiniana, Cladosporium sphaerospermum, P. chrysogenum* (SPT: 1:20 w:v; IDT: 1,000 PNU/mL), mold mix 2: *Aureobasidium pullulans, Drechslera spicifera, Fusarium moniliforme, Mucor plumbeus, Rhizopus stolonifer* (SPT: 1:40 w:v), IDT (1,000 PNU/mL), mold mix 3: *A. alternata, A. niger, C. sphaerospermum, P. chrysogenum* (SPT: 1:20 w:v), IDT (1,000 PNU/mL), weed mix: cocklebur, lamb’s quarter, rough/redroot pigweed (SPT: 1:20 w:v), IDT (1,000 PNU/mL), grass mix: meadow fescue, Kentucky blue/june, orchard, redtop, perennial rye, sweet vernal, timothy (SPT: 1:20 w:v), IDT (1,000 PNU/mL), tree mix: American beech, American/Eastern sycamore, American elm, black walnut, black willow, Eastern cottonwood, Red oak, red/river birch, shagbark hickory, sugar/hard maple, white ash (SPT: 1:20 w:v), IDT (1,000 PNU/mL)	Subjective Scoring: A scale from 0 to 4 was used to consider erythema, wheal size, turgor, and depth of induration. Positive reaction: if the score was ≥2 compared to histamine (positive control) and saline solution (negative control). Objective: MWD = average length and width of the wheel. Positive reaction: if the MWD exceeded the threshold value (the mean MWD of the positive and negative controls)	Compared with IDT (gold standard): SPT Sens 47.0%. Spec: 92.1%. PPV: 36%. NPV: 95%. Agreement (Cohen’s kappa): 0.424 (moderate), false negative rate in allergen mixtures possibly due to dilution of individual components. Future studies should test individual allergens, thus increasing the reactivity in SPT and IDT. The low sensitivity of SPT limits its usefulness in CAD

BU, Bethesda unit; CAD, Canine atopic dermatitis; CI, Confidence interval; DF, *Dermatophagoides farinae*; DP, *D. pteronyssinus*; IDT, Intradermal skin test; MWD, Mean wheal diameter; PNU, Protein nitrogen unit; SPT, Prick test; Sens, sensitivity; Spec, Specificity; w/v; y, Years; NR, Not reported; The samples were weighed per volume.

**Figure 2:**
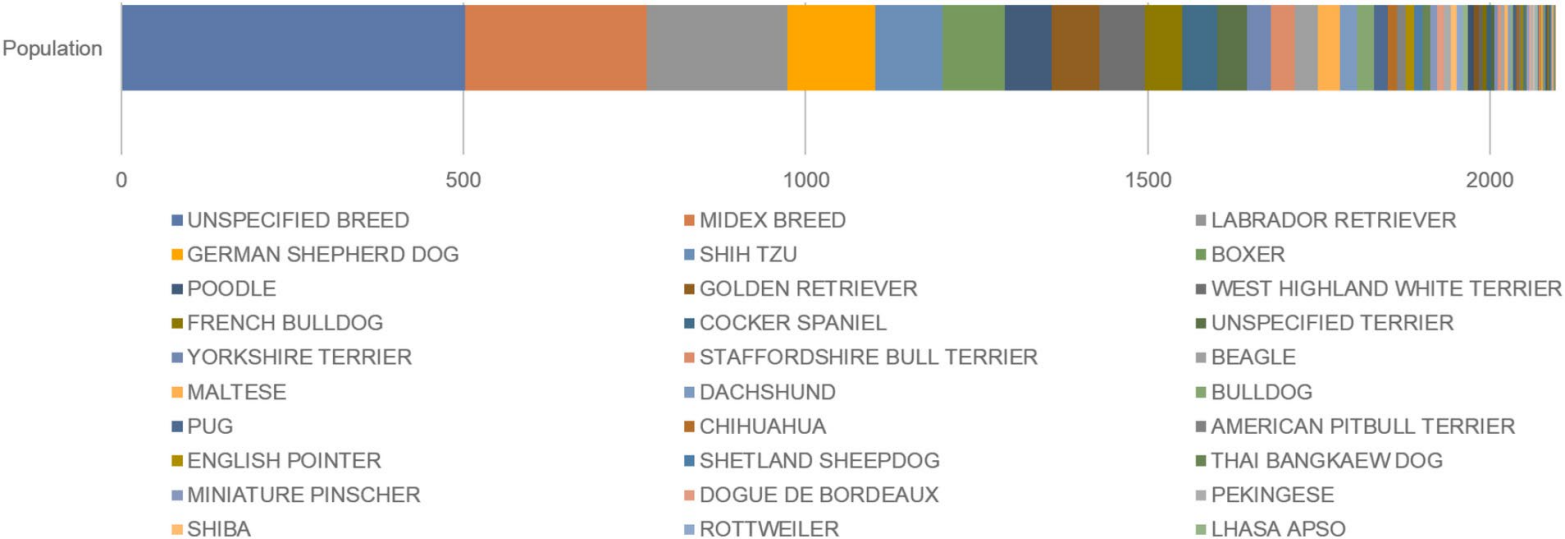
Distribution of dogs in the selected studies according to breed. Nomenclature according to the *Fédération Cynologique Internationale* (https://www.fci.be/en/nomenclature).

Tables 1–4 display the information extracted from the four diagnostic test groups. Since 2010, Favrot’s clinical criteria (14) have been applied in 41.6% of studies (30/72); before this report, there was no consensus on the case definition for CAD. The five most represented breeds included mixed, Labrador, German Shepperd, Shih Tzu, and Boxer. However, the breed of almost 500 out of 2,096 dogs was not specified. All remaining and well-known dog breeds are represented in Figure 2. Table 1 presents a detailed feature of selected articles reporting a serologic-specific allergen test (SAT) for CAD diagnosis. The Sensitivity, Specificity and Positive or Negative predictive comparison values between studies that met the criteria for these analyses are presented in Table 5. Finally, the available data for allergen prevalence according to allergen reactivity of CAD patients against mites, insects, trees, and molds, is presented in Table 6.

**Table 4:** The patch test was used for the diagnosis of canine atopic dermatitis (*n* = 1).

Cons.	Ref.	Country	Sample size (n) and Sex	Breed (n)	Age	Favrot’s criteria	Allergen (concentration)	Cutoff point to positivity	Relevant findings
1	(81)	USA	6 Males (3), females (3)	Maltese, Beagle (unspecified)	3.8–12.8 y (median: 6.5 y)	Not	DF (500 μg per patch)	Observation of macroscopic and microscopic inflammatory reactions at the patch application site: Positive reactions: Macroscopic inflammation: Visible erythema, edema, induration, and vesiculation at the patch application site. Microscopic inflammation: Increased infiltration of eosinophils and CD3+ T lymphocytes in skin biopsies. Higher scores for epidermal and dermal inflammation	The Viaskin® epicutaneous system induced stronger inflammation in HDM-allergic dogs than Finn chamber® patches. Different reactions to allergen and control patches were significant only with Viaskin®—macroscopic cutaneous observations correlated with microscopic inflammation. Viaskin® may be a more effective tool for diagnosis and potentially for immunotherapy in CAD

CAD, Canine atopic dermatitis; y, Years; HDM, House dust mite; The samples were weighed per volume.

**Table 5:** SAT (specific allergen serological test) comparison table.

SAT TEST	(Sens)	(Spec)	(PPV)	(PNV)	Cutoff point (positive)
ELISA IgE. Ginel et al. (44)	72.23%	41.6%	76.47%	35.71%	OD >0.15
ELISA CMG Immunodot IgE. Mueller et al. (46)	90%	81%	NR	NR	NR
ELISA FcεRI α IgE (DF, DP). Foster et al. (49)	67.9%; 19.3%	89.3%; 96.6%	NR	NR	OD >0.250
ELISA IgE. Sævik et al. (26)	53.6%	84.4%	75%	67.5%	OD >0.250
ELISA FcεRI α IgE (DF, DP). Popiel and Cekiera (56)	82.3%; 65.1%	27.2%; 80%	NR	NR	50 EAU

EAU, ELISA absorbance; PPV, Positive predictive value; PNV, Negative predictive value; Sens, Sensitivity; Spec, Specificity; NR, not reported.

**Table 6:** Table of allergen prevalence according to allergen reactivity in canine atopic dermatitis.

Allergen	Mites						Insects				Trees			Molds
Reference	DF	DP	Mix	*B. tropicalis*	*TP*	House dust	*Peristolaca americana*	Insect mix	Ants’ mix	Flea	Mix Epithelia	*C. dactylon*	*B. mutica*	Polen-mix grass
Chanthick et al. (83)	75%	54%		NR	NR	26%	24%	NR	NR	NR	NR	NR	22%	NR
Yang Sung and Pi Huang (84)	70%	70%	68%	NR	NR	72%	NR	33%	NR	NR	NR	NR	NR	NR
Taszkun (85)	94%	NR	71%	NR	NR	NR	NR	NR	NR	4%	4.15%	NR	NR	20%
Pereira et al. (57)	33%	NR	NR	49%	57%	NR	NR	NR	NR	NR	NR	42%	NR	NR
Chermprapai et al. (23)	65%	59%	48%	NR	NR	36%	NR	NR	NR	NR	NR	NR	NR	NR

DF, *Dermatophagoides farinae*; DP, *D. pteronyssinus*; NR, not reported; TP, *Tyrophagus putrescentiae*.

## Discussion

This systematic review describes the available literature on factors related to the diagnosis of allergens associated with CAD, a disease of complex etiology. Identifying specific clinical criteria is the cornerstone of CAD diagnosis. To identify allergens related to CAD provides the clinician with additional therapeutics to offering accurate therapeutic plans to their patients. Diagnostic tests with high reliability, specificity, repeatability, and sensitivity are needed as diagnostic alternatives for clinicians to achieve a more accurate diagnosis of the allergens involved. One of the most critical findings of this work was the failure to find a gold standard parameter. Another limitation in evaluating diagnostic performance is the heterogeneous concentrations of allergens used in dermabrasion tests, mode of preparation, and source of extraction.

For the SPT, which has been used regularly for several years in the diagnosis of allergens in human medicine, only two studies have evaluated the diagnostic performance of CAD in case-control studies. The findings of the two studies are contradictory, so SPT does not have enough references to judge its accuracy, making it necessary to conduct more studies on the subject since, owing to its easy design and application, it could be a useful tool in diagnosing allergens responsible for CAD crises. On the other hand, the measurement of serum IgE concentrations, which is often widely accepted in human AD allergen diagnosis, also presents a range of variations as complementary diagnoses of CAD. In addition, their results must be analyzed in the context of the patient’s clinical signs at a given time. The same doubts exist about IgE testing, as no evidence-based medical criteria were found. In this context, human dermatology has achieved an important consensus for diagnosing allergens associated with AD, but we still have a long way to go in CAD.

This systematic review identified 72 studies published between 1982 and 2023 in which microbial and nonmicrobial allergens were identified. In addition to IDT, SPT, the patch test, and ST, the study by Sævik et al., a combination of ELISA and intradermal injections of allergens to detect Ig-E-mediated reactivity in atopic dogs, revealed no concordance between serological and dermal tests and IgE positivity in the diagnosis of allergens related to CAD (26). Sasaki et al. used a crude allergen extract from Japanese cedar pollen to assess reactivity against it via IDT in dogs (27).

The results of this systematic review highlight the actual limitations in defining the utility of diagnostic tests to identify allergens in CAD patients. This discussion has taken place in the medical and scientific community, and the results of our systematic review did not answer the research question, which was intended to identify diagnostic tests that could be considered consistent, reliable, sensitive, and specific for CAD.

The role of immunoglobulins in the pathogenesis and diagnostic criteria for CAD has not been fully elucidated (28). Mueller et al. reported no statistically significant differences in the IgA concentration in skin washings between atopic and clinically healthy dogs (28). In contrast, Noli et al. reported a lack of IgE reactivity of serum from atopic dogs against *Dermatophagoides pteronyssinus*-derived allergens (Der p I, Der p II), and *D. farinae* antigens Der f I, and Der f II, which seem unlikely to be major allergens in dogs with CAD. However, the serum of atopic dogs reacts to a 90 kDa polypeptide of *D. farinae*, according to Western blot analysis (29). Hämmerling, evaluating IgE and the IgGa subclass in atopic dermatitis dogs, concluded that finding DP- and DF-related IgE renders this IG isotype more relevant than IgGa in detecting DA-related allergens (30).

Willense et al., and Moya et al. reported IgE and the IgG 1, IgG 2 subclasses against fractions of Der f 2 and Der f 1, Der f 18 in a lesser proportion; the IgG1 subclass seems to distinguish both IgE and House Dust Mites Allergens (HDMs) in CAD (31, 32). IgE reactivity against HDMs is more common than IgG reactivity in CAD (33). However, several factors must be considered when interpreting the results that affect their titer. Day and Corato reported heterogeneous IgG subclass reactivity against the most common allergens related to atopic dermatitis in dogs (34). Interestingly, helminth parasite infestation stimulates IgG production against IgE epitopes and decreases their levels in atopic dogs concomitantly infected by helminths and acari (35). Some patterns of IgE sensitization in dogs have been described in humans (e.g., Der f 18) (35), and the positive results of dermabrasion tests with concordant allergy-specific IgE levels in atopic dogs in Brazil are evidenced by the high prevalence of dust mites in Sao Paulo (24). For some authors, total IgE tests are unreliable and do not differentiate CAD (36). Jang et al. reported no significant differences in allergen reactivity between IDT and SAT (37).

Cross-reactivity has been reported in CAD dogs against plant allergens (38) and HDMs (DF and DP) (39), as well as with invertebrates and storage mites (*Tyrophagus putrescentiae*) with DF (40). Dogs sensitized to grass pollen often react to other allergens, particularly DF (24, 38, 39), and there is apparent sensitization between related allergen groups (house dust/storage mites, epidermis/fibers, trees, weeds, grass pollen, and molds) (41, 42).

The predominant sensitization against HDMs was common in several seasonal and nonseasonal countries reported from 1983 to 2023 (1–26, 28–31, 33–40, 43–76), indicating a consensus on a higher prevalence of HDMs than other allergenic groups, followed by plant pollen (39), animal allergens and molds, except for the study by Jung Kim et al., which described a higher prevalence of mold (77) (related to closed spaces, climatic changes that can influence findings).

Allergens have been described in HDMs, especially DFs, with high and low molecular weights classified as major and minor allergens according to their allergenic potential. Among the major allergens, the following have been reported: chitinase (Der f15) (48) and Der f 18, with greater sensitization in CAD (84.6%) than in human DA (50%) (48). Minor: In the Der 1 and 2 groups, approximately 50% of CAD dogs were sensitized to Der 1 and Der 2 in a study in Japan (40), and little importance in CAD has been reported (47), similar to the Der p I, Der p II, Der f 1, and Der f 2 (48) fractions. They were also described as irrelevant in the study by Noli et al. (29). Der f 1 is the major allergen in humans, and Der f 2 in SAT in CAD is poorly recognized by IgE (53). Other studies reported a high prevalence (86.7% in humans and 94.1% in dogs) (40). Khantavee et al. reported five major DF fractions (Der f Alt a 10, EF1-*α*, the gelsolin-like allergen Der f 16, Der f 28 and Der f 2) and Der f 3, Der f 10, Der f 20, and Der f 32 as minor allergens (33). Exposure to furniture and textiles has been associated with DF sensitization via the Zen 1 fraction, which strongly correlates with the crude DF extract in the SAT (62).

Geographical variations accounting for the prevalence and diversity of allergens (humidity and temperature in HDMs) should be taken into account (67), reflecting exposure factors (50). There are allergens specific to each region, including Japanese cedars (JCs) and pollen in Japan (38). *Rumex acetosa* (sorrel) in Italy (61), the pollen of some weeds in Thailand (23), and *Cynodon dactylon* in Brazil (57). In addition, environmental and surrounding conditions increase the prevalence of certain storage mites, as reported in Portugal (65), Brazil (*T. putrescentiae, Lepidoglyphus destructor,* and *Blomia tropicalis*) (53), and Thailand (23), where IgE titers increased during the rainy season (23). Air currents affect pollen distribution (39), although indoor aeroallergen deposition is independent of seasonality (42).

There are discrepancies between SATs (43) and various IgE measurement techniques lacking quality control and providing different results when the same sample is analyzed simultaneously with other equipment and methods (59). There is intra- and interlaboratory variability, so a quality assurance program (reliability) is needed (52).

Thirty-seven out of the 72 articles evaluated were related to IDT: only five studies reported sensitivity and specificity measurements (Table 5). Considering the positivity cutoff point as a determinant of these values is important. The positivity cutoff points for SAT do not have a consensus, ranging from OD >0.15 (44) to OD >0.250 (26, 49); other reported 50 EAU (56), or units were not reported (46).

Lowering cutoff point arbitrarily increases the probability of false positives (decreasing the test’s specificity). On the contrary, raising cutoff point affects the sensitivity and the possibility of false negatives. Standardizing this evaluation according to the techniques used and the allergens evaluated (allergenic potential) is essential (26). Cross-reactivity should be reduced with inhibitors of carbohydrate cross-reacting determinants (CCDs) (60). The study of allergenic fractions with allergenic potential detected by specific IgE would increase the effectiveness and greater reliability of the tests, mitigating false positives. IgG1, which reacts against DF and DP, was also reported as the predominant IgG subtype in a set of atopic dogs (33).

Threshold concentrations (positive nonirritating reactions) in IDT in dogs have been reported to be similar to those in humans (66). Standardized extracts for human medicine are often effective in CAD (78). Some studies define thresholds for certain allergens (values expressed in Protein Nitrogen Units, PNU): grasses, weeds, trees, molds and insects (1,750 PNU/mL), flea allergens (1:500 w/v), DPs (250 PNU/mL), DFs and *T. putrescentiae* (100 PNU/mL), epidermis (all at least 1,250 PNU/mL), human dander (300 PNU/mL), the optimal concentration of histamine (positive control) at 1:10,000 w/v. (76), Irritability at 1:10,000 w/v for DF and DP; 1,000 PNU for dust (36); and highly concentrated pollen extracts of 1,000–1,500 PNU (66). It has also been shown that mixed allergen extracts (26) in HDMs and house dust result in false negatives and low sensitivity and specificity. Individual DF and DP extracts, not house dust, are indicated (75).

IDT usually exhibits high sensitivity and specificity in detecting allergens in dogs with CAD (26). However, these values may vary depending on the allergen and concentration used. There is no clear consensus on the optimal allergen concentrations for IDT, which may affect the accuracy and reproducibility of the results. Some studies have evaluated different allergen concentrations and reported that higher concentrations may increase sensitivity and the risk of false positive reactions (36, 76). Interpretation of IDT results can be subjective and can vary among raters. Scales from 0 to 4 assess papule size, turgor, and erythema. Standardizing interpretation methods to reduce variability and improve comparability between studies and clinical settings is crucial.

Most of the works found in this systematic review reported prevalence values of mixtures or individual allergens without homogeneity in concentrations on the basis of the definition of a case or disease as CAD. The current clinical criteria were established in 2010 by Favrot (14), who issued eight clinical criteria, more than five of which had a sensitivity of 85% and a specificity of 80%. Assuming these criteria as a case definition, the previous prevalence would be reconsidered (because of the possibility to have been included nonatopic individuals), and therefore, false-positive results would have been obtained (Table 5).

Cross-reactivity between related allergens can complicate the interpretation of IDT results (38, 39, 42). Identifying and considering these factors when allergens are selected for testing are important. Co-sensitization to multiple allergens is common in dogs with AD (41), which may influence allergen choice (67). Allergen prevalence can vary significantly by geographic region, highlighting the importance of tailoring allergen panels to local conditions. Factors such as humidity and temperature can affect allergen sensitivity and should be considered when interpreting IDT results (61).

Standard protocols for performing and interpreting the IDT, including allergen concentration and interpretation criteria, are needed. Combining subjective and objective methods may yield more reliable IDT results (79). Longitudinal studies have been conducted to assess the long-term effectiveness of IDT and IDT-based immunotherapy. The inclusion of new relevant allergens in different geographic regions should be investigated to improve the accuracy and utility of IDT.

The study by Carmona et al. revealed a moderate sensitivity of 66% for SPT and a high specificity of 100% (80), in parallel with the study by Fleischman and Morris (25), where the intradermal test (IDT) and the SPT were compared, with a low sensitivity of 47% and a high specificity of 92.1% for the latter. SPT does not require sedation, is less invasive and comfortable for the animal, is less expensive than IDT, and has high specificity, reducing the probability of false positives. It appears to have moderate to low sensitivity, limiting its usefulness. However, the low reactivity to allergen mixtures (used in both studies) due to the dilution of individual components must be considered, which can result in false negatives. The interpretation of the results can be subjective and vary between evaluators, as with IDT. The agreement between the SPT and the IDT in the Fleischman and Morris (25) study was moderate (Cohen’s kappa value = 0.424), suggesting that both methods may complement each other. Studies should be conducted in larger and more diverse populations to validate the findings and improve the generalizability of the results with individual allergens instead of mixtures by increasing the reactivity and accuracy of the SPT; additionally, protocols for the performance and interpretation of the SPT should be developed and standardized to reduce variability and improve the comparability of results. Panels of new allergens relevant to different geographical regions should be included (25).

In the study by Olivry et al., an extract of *D. farinae* was used (500 μg per patch) (81). The presence of visible erythema, edema, induration, and vesiculation at the application site was evaluated. Macroscopic observations of skin inflammation were compared with microscopic observations. The study was conducted with a small, nonrepresentative sample. We wondered whether individual extracts should be used instead of mixtures with different standardized concentrations, considering irritant thresholds. Studies should be designed with more individuals and diverse races to validate the findings and compare the system evaluated with other diagnostic and treatment methods to evaluate its relative effectiveness.

In the study by Sævik et al., intradermal injections of sera from allergen-positive dogs were performed via ELISA. On the other hand, the challenge test involves the controlled administration of a specific allergen to the patient to observe whether an allergic reaction occurs (26), using a crude allergen extract from Japanese cedar pollen in dogs. The challenge test fell into disuse owing to the risk of adverse reactions and has been replaced by dermabrasion tests (27).

Given the gaps in knowledge, this manuscript proposes bringing together world experts in veterinary allergology to agree on critical points and issuing a general guide as a starting point for new research with internal and external validity, reproducibility, and comparative capacity. It is necessary to clearly and reliably define prevalence tables and statistical association measures to support the diagnosis of CAD. We urge the execution of studies with significant sample sizes, diverse racial groups, and sexual parity, considering the factors mentioned earlier, such as the environment, seasonality, humidity, temperature, and geographic variations in the prevalence of allergens (67).

## Advantages and limitations of systematic review

Our systematic review has several strengths. We followed a structured approach anchored in a clearly defined research question previously documented and validated by systematic reviews from health field experts. Our extensive literature search spanned numerous sources, including general databases, search engines, journals, and conference proceedings, enabling us to capture data as far back as 1949. By excluding geographic and time-based limitations, we aimed to reduce potential biases. Additionally, the extracted information was rigorously organized; one author prepared a matrix of findings, which a second author subsequently reviewed to ensure accuracy across studies of varying quality and methodology. As a limitation, 21 relevant documents identified through abstract screening were not available as open access, so they could not be fully processed as complete texts to determine their definitive relevance, and we did not consider gray literature. To mitigate this, we employed snowballing techniques.

Finally, this manuscript presents a detailed, comprehensive, and transparent systematic review of allergen diagnostic methods for CAD, following PRISMA standards. The methodology used in the review, including exhaustive search and content analysis with Atlas TI software, ensures the robustness and reproducibility of the findings. This includes the evaluation of multiple databases and the application of rigorous inclusion and exclusion criteria. Different diagnostic methods are compared, providing a clear overview of their advantages, limitations, and relative effectiveness. We report sensitivity and specificity data from relevant studies, which helps the scientific community understand the gaps in knowledge and scientific rigor to improve the accuracy of these diagnostic methods. The most common allergens used in the studies were identified and reported, which could guide future research. We highlight the application of Favrot’s clinical criteria in 41.6% of the studies since 2010 (14), highlighting the importance of these criteria in defining CAD cases. We identified gaps in current knowledge that suggest the need for future research to standardize allergenic extracts, define cutoff points in serological tests, and consider environmental, geographic, and demographic variables. These findings are valuable to the scientific community, as they provide a solid basis for improving the diagnosis and management of CAD and guiding future research in this field.

## Conclusion

Even though most scientist and practitioners consider IDT as the standard test to diagnose allergen sensitization in CAD patients, our results suggest caution must be kept when considering the available tests as a gold standard to identify allergen sensitization in dogs affected by CAD. Current methods have advantages and limitations. There is no consensus on antigen concentrations, no standardized cutoff points for SAT positivity have been defined, and there is no laboratory regulation or monitoring. Favrot’s clinical criteria have been applied in 41.6% of studies since 2010, highlighting their importance in defining CAD cases. A total of 136 different allergens used in the studies were identified, providing a basis for future research and clinical practice. The development and standardization of allergenic extracts for diagnostic testing are crucial, ensuring the consistency and comparability of results between different studies and laboratories. Studies that consider environmental, geographic, and demographic variables should be conducted to better understand how these factors affect the prevalence and severity of CAD. We urge further studies to evaluate the efficacy and applicability of the Favrot’s criteria in different populations and settings. Longitudinal studies should be conducted to better understand the progression of CAD and the long-term effectiveness of different diagnostic methods and treatments and to further explore co-sensitization and cross-reactivity between different allergens to improve the accuracy of diagnostic tests and the formulation of specific immunotherapies. Investigate the impact of CAD on the quality of life of dogs and their owners and how different diagnostic methods and treatments can improve these aspects. These proposals may help advance the knowledge and management of CAD, improving the diagnosis and treatment of this condition.

## Data Availability

The raw data supporting the conclusions of this article will be made available by the authors without undue reservation.

## Footnotes

^1^ https://wavd.org/

## Glossary

AD: Human Atopic Dermatitis
ASIS: Allergen-specific IgE serology
BU: Bethesda Unit
CAD: Canine Atopic Dermatitis
CADESI: Canine Atopic Dermatitis Extent and Severity Index
CCDs: Carbohydrate Cross-Reacting Determinants
CMG Immunodot: ELISA test using allergen-specific anti-canine IgE monoclonal antibodies.
Der f 15, Der f 18: *Dermatophagoides farinae*-derived Major allergens. Each one are the major allergens of the American house dust mites.
Der f 1, Der f 2, Der f 18: *Dermatophagoides farinae*-derived Minor allergens. Each one are the major allergens of the American house dust mites.
Der p I, Der p II,: *Dermatophagoides pteronyssinus*-derived allergens (major allergens of the American house dust mites).
DF: *Dermatophagoides farinae*
DP: *Dermatophagoides pteronyssinus*
EAU: ELISA Absorbance Units
ELISA: Enzyme-Linked Immunosorbent Assay
HDM: House Dust Mites Allergens
IDT: Intradermal Test
IgE: Immunoglobulin type E
IgG: Immunoglobulin type G
IgG1: Immunoglobulin type G subclass 1
IgG2: Immunoglobulin type G subclass 2
MWD: Mean Wheal Diameter
NE: Nitrogen Equivalents
OD: Optical Density
PNU: Protein Nitrogen Units
PNV: Predictive Negative Value
PPV: Predictive Positive Value
PVAS: Pruritus Visual Analog Scale
SAT: Serologic-specific Allergen Test
SD: Standard Deviation
Sens: Sensitivity
Spec: Specificity
SPT: Skin Prick Test
w/v: Weight-to-volume Ratio
